# Tracing the molecular route to progression in miRNA-biogenesis-defective thyroid lesions

**DOI:** 10.1172/jci.insight.198338

**Published:** 2026-02-09

**Authors:** Anne-Sophie Chong, Carla Roca, Paula Morales-Sánchez, Eduard Dorca, Verónica Barea, Ignacio Ruz-Caracuel, Pablo Valderrabano, Carlota Rovira, Cristina Jou, Dorothée Bouron-Dal Soglio, Rebecca D. Chernock, Giovana T. Torrezan, Marc Pusztaszeri, José M. Cameselle-Teijeiro, Xavier Matias-Guiu, Clara V. Alvarez, Héctor Salvador, Jonathan D. Wasserman, Luis Javier Leandro-García, William D. Foulkes, Eduardo Andrés-León, Paula Casano-Sancho, Barbara Rivera

**Affiliations:** 1 Program in Molecular Mechanisms and Experimental Therapy in Oncology (Oncobell), Bellvitge Biomedical Research Institute (IDIBELL), L’Hospitalet de Llobregat, Barcelona, Spain.; 2 Genetics Program, Faculty of Biology, and; 3 Department of Biomedical Sciences, Faculty of Medicine and Health Sciences, University of Barcelona, Barcelona, Spain.; 4 Pathology Department, Bellvitge University Hospital, L’Hospitalet de Llobregat, Barcelona, Spain.; 5 Genetics and Genomics, Faculty of Biology, University of Barcelona, Barcelona, Spain.; 6 Ramón y Cajal Health Research Institute (IRYCIS), Ramón y Cajal University Hospital, CIBERONC, Madrid, Spain.; 7 Department of Pathology, Ramón y Cajal University Hospital, Madrid, Spain.; 8 Department of Endocrinology and Nutrition, Hospital Universitario Ramón y Cajal, IRYCIS, Madrid, Spain.; 9 Department of Pathology, Hospital Sant Joan de Déu, University of Barcelona, Barcelona, Spain.; 10 Centre Hospitalier Universitaire Sainte-Justine Research Center, Université de Montréal, Montréal, Quebec, Canada.; 11 Department of Pathology and Immunology, and; 12 Department of Otolaryngology Head and Neck Surgery, Washington University School of Medicine, St. Louis, Missouri, USA.; 13 Clinical and Functional Genomics Group, International Research Center/CIPE, A.C. Camargo Cancer Center, São Paulo, Brazil.; 14 Department of Pathology, Jewish General Hospital, McGill University, Montreal, Quebec, Canada.; 15 Department of Pathology, Clinical University Hospital of Santiago de Compostela, Health Research Institute of Santiago de Compostela (IDIS), University of Santiago de Compostela, Santiago de Compostela, Spain.; 16 Department of Pathology, Hospital Universitari Arnau de Vilanova, Universitat de Lleida, IRBLLEIDA, Lleida, Spain.; 17 Neoplasia & Endocrine Differentiation P0L5, Centre for Research in Molecular Medicine and Chronic Disease (CIMUS), Santiago de Compostela, Spain.; 18 Department of Oncology, Hospital Sant Joan de Déu, University of Barcelona, Barcelona, Spain.; 19 Division of Endocrinology, Department of Paediatrics, The Hospital for Sick Children, University of Toronto, Toronto, Ontario, Canada.; 20 Hereditary Endocrine Cancer Group, Human Cancer Genetics Program, Spanish National Cancer Research Centre (CNIO), Madrid, Spain.; 21 Department of Human Genetics, and; 22 Gerald Bronfman Department of Oncology, McGill University, Montreal, Quebec, Canada.; 23 Bioinformatics Unit, Institute of Parasitology and Biomedicine López-Neyra (IPBLN), CSIC, Granada, Spain.; 24 Pediatric Endocrinology Department, Institut de Recerca Sant Joan de Déu, University of Barcelona, Barcelona, Spain.; 25 Centro de Investigación Biomédica en Red de Diabetes y Enfermedades Metabólicas Asociadas (CIBERDEM), Instituto de Salud Carlos III, Madrid, Spain.

**Keywords:** Endocrinology, Genetics, Oncology, RNA processing, Thyroid disease, Transcriptomics

## Abstract

Germline and somatic changes in *DICER1* and *DGCR8* microprocessors confer risk of developing benign and malignant thyroid lesions, yet the molecular events driving malignant transformation remain unclear. We trace the molecular trajectories from benignity to malignancy in *DICER1-* and *DGCR8*-mutated thyroid lesions using multiomic profiling on over 30 *DICER1-/DGCR8*-mutated samples. Our findings reveal a progressive, specific, and linear accumulation of genetic changes, which when combined with enhanced downregulation of miRNAs distinguished *DICER1-/DGCR8*-malignant lesions from their benign counterparts. Compensatory hypomethylation of miRNA-encoding genes characterized *DICER1-/DGCR8*-benign lesions, but as the tumors progressed to malignancy, methylation was partly reimposed, reversing the attempts to activate miRNA-encoded genes and further compromising miRNA production. Transcriptomic analyses revealed mutation-specific effects on the microenvironment, whereby *DICER1* mutations activated canonical thyroid cancer progression pathways, whereas altered *DGCR8* associated with immune-related changes. This work unveils specific molecular events underlying malignant progression of miRNA-biogenesis-related thyroid tumors and identifies potential biomarkers and disease etiology mechanisms.

## Abstract 2

Multiomic analysis in *DICER1*/*DGCR8*-thyroid lesions depicts the benign to malignant progression route at the genomic, miRNAomic, and transcriptional levels and identify an epi-signature of *DICER1*-cancers.

## Graphical abstract

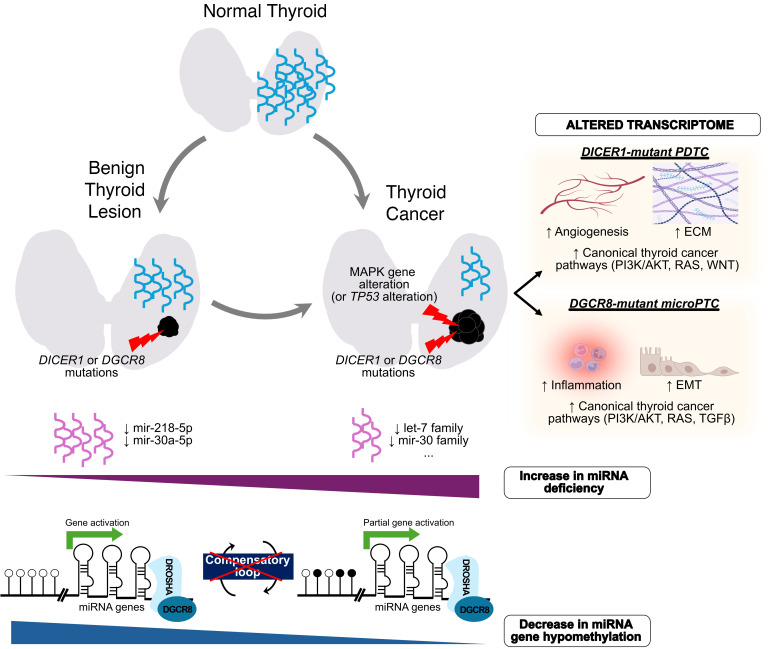

## Introduction

MicroRNAs (miRNAs) are small regulatory RNAs of 22 nucleotides processed by the miRNA biogenesis machinery. In humans, over 2,300 miRNAs are organized into families with tissue- and time-specific expression patterns (1, 2). There is redundancy in the miRNA targeting maintaining tight regulatory loops that ensures the proper expression of transcripts (3, 4). In the canonical miRNA biogenesis pathway, the transcription of a primary microRNA (pri-miRNA) by RNA polymerase II occurs in the cell nucleus leading to a hairpin shaped pri-miRNA that is cleaved by the nuclear microprocessor (DROSHA-DGCR8 complex) into a precursor miRNA (pre-miRNA) of approximately 70 nucleotides. The pre-miRNA is then exported to the cytoplasm where DICER1 cleaves the apical loop to generate a double-stranded miRNA. The passenger strand will then be degraded and the mature canonical miRNA is loaded into the RISC complex to bind to its target mRNA and inhibit translation (5). In the noncanonical pathway, intron-encoded miRNAs (mirtrons) are first processed by the spliceosome during the transcription of the encoding mRNA. As a result, a microprocessor-independent pre-miRNA is formed and further cleaved by DICER1. Other noncanonically processed miRNAs exist, adding diversity and complementarity to miRNA-regulated translation (6).

Germline pathogenic variants (GPVs) in *DICER1* and *DGCR8* are linked to tumor susceptibility (7). *DICER1*-related tumor predisposition (DRTP) (OMIM #601200) is a pleiotropic cancer predisposition disorder caused by GPVs in *DICER1* that compromise its miRNA biogenesis function. Clinical manifestations of DRTP are highly variable, ranging from benign tumors to low-incidence malignancies, primarily affecting children and young adults (8). Among the various phenotypes, thyroid follicular nodular disease (TFND) represents the most prevalent condition in DRTP patients. In 2011, *DICER1* was identified as the first putative gene responsible for susceptibility to TFND (9), with subsequent studies reporting a 16-fold higher risk of differentiated thyroid cancer (DTC) in *DICER1* carriers (10). Additional reports have highlighted the presence of *DICER1* mutations in benign thyroid lesions (BTLs), including TFND and follicular thyroid adenomas (FTAs), as well as in noninvasive follicular thyroid neoplasms with papillary-like nuclear features (NIFTPs) and various DTC subtypes, such as follicular thyroid carcinoma (FTC), follicular variant of papillary thyroid carcinoma (FVPTC), and classic papillary thyroid carcinoma (CPTC) (11). Poorly differentiated thyroid carcinomas (PDTCs) are aggressive and often fatal tumors that have also been associated with identical *DICER1* mutations to those driving indolent miRNA-associated thyroid nodules (12), thus precluding their prognostic utility. This underscores the need to identify changes specific to malignant transformation that may be clinically actionable for improving patient management.

Our group previously identified a germline variant in *DGCR8* conferring susceptibility to familial TFND and peripheral schwannomatosis (13). Subsequent reports revealed the presence of the same *DGCR8* mutation in 1 patient with TFND and DTC (14), 4 widely invasive FTCs (15, 16), 1 minimally invasive encapsulated FVPTC (15), and 1 PDTC (17), confirming the role of *DGCR8* mutations in the development of benign and malignant thyroid tumors, particularly in follicular-patterned lesions. Thus, the involvement of these 2 main miRNA biogenesis genes, *DICER1* and *DGCR8*, in thyroid tumorigenesis emphasizes the functional relevance of miRNA regulation in the development of the thyroid gland and provides an opportunity to uncover key effectors in miRNA-driven cancer development.

Most *DICER1*-associated tumors harbor biallelic changes, where one allele carries a loss-of-function (LoF) variant, either inherited or acquired, while the other allele harbors an acquired somatic mutation affecting the RNase IIIb catalytic domain. These somatic mutations cluster at specific hotspot residues that are essential for metal-ion binding and miRNA processing. In contrast, LoF variants are scattered throughout the gene (7, 18). In *DGCR8*-mutated thyroid lesions, a distinct hotspot mutation (E518K) located in the RNA binding domain of DGCR8 in combination with loss of heterozygosity (LOH) has been reported (7, 13), along with co-occurrence of *RAS* mutations in the thyroid cancers (15, 16, 19). In *DICER1* cancers, mutations were mutually exclusive with canonical MAPK pathway (*BRAF* and *H-/K-/N-RAS*) changes and receptor tyrosine kinase fusions (*RET*, *NTRK*, and *ALK*) (12, 20–23). Despite this difference, both *DICER1*- and *DGCR8*-mutated thyroid tumors are predominantly follicular patterned (15, 24, 25) and proposed to belong to the transcriptionally *RAS*-like subtype (26), yet the processes downstream of the miRNA biogenesis deficiency that lead to thyroid cancer remain understudied.

Seeking to expose the specific molecular routes distinguishing benign tumors from malignant ones, we undertook an integrative characterization of the molecular landscapes in BTLs and malignant thyroid lesions with *DICER1* and *DGCR8* mutations. By integrating genomic, methylomic, miRNAomic, and transcriptomic data from mutated tumors, we delineate distinct and shared trajectories of benign and malignant transformation. Our findings offer insights into miRNA-driven thyroid tumorigenesis and the molecular events underlying disease progression.

## Results

### Identification of DICER1- and DGCR8-mutated thyroid samples.

We genotyped 581 thyroid lesions from 425 patients for 31 single nucleotide variants in miRNA biogenesis genes. Screening identified *DICER1* hotspot mutations in 9 of 63 (14.2%) pediatric samples corresponding to 7 patients (Supplemental Table 1; supplemental material available online with this article; https://doi.org/10.1172/jci.insight.198338DS1), all with follicular-patterned thyroid lesions (Supplemental Methods). Four mutated samples were from *DICER1* GPV carriers, 2 were sporadic, and 1 was found to harbor a mosaic variant. The remaining 2 samples (FTA and FTC) belonged to a single patient in which we did not identify a second LoF hit by whole-exome sequencing (WES).

In the adult series, we identified *DICER1* hotspot mutations in 3 out of 518 (0.58%), including (a) 1 TFND sample (44 years old, female) harboring a *DICER1*, c.5439G>T, p.E1813D hotspot mutation (no LOH), likely to be sporadic, as a paired TFND from the same patient did not carry any hotspot mutation; (b) 1 FTC; and (c) 1 PDTC, for which normal tissue was not available. The *DGCR8* E518K hotspot mutation was found in 1 out of 518 (0.19%) (FTC sample confirmed to be sporadic; Supplemental Table 1). An extra group of 27 previously identified thyroid lesions from 27 patients with *DICER1* or *DGCR8* changes was also collected for subsequent omic analysis (Table 1 and Supplemental Figure 1). Supplemental Table 2 contains clinicopathological details for all *DICER1-*/*DGCR8*-mutated thyroid lesions included.

### Genomic landscape of DICER1- and DGCR8-mutated BTLs and malignant thyroid lesions.

We first performed WES on 18 samples harboring *DICER1*/*DGCR8* mutations and integrated the data with published cases (12–14) and cases from The Cancer Genome Atlas Thyroid Cancer (TCGA-THCA) project (19). In total, 18 *DICER1*-mutated thyroid cases (5 benign, 13 malignant) and 10 *DGCR8*-mutated thyroid cases (6 benign, 4 malignant) were analyzed (Table 1 and Supplemental Table 3).

**Figure 1:**
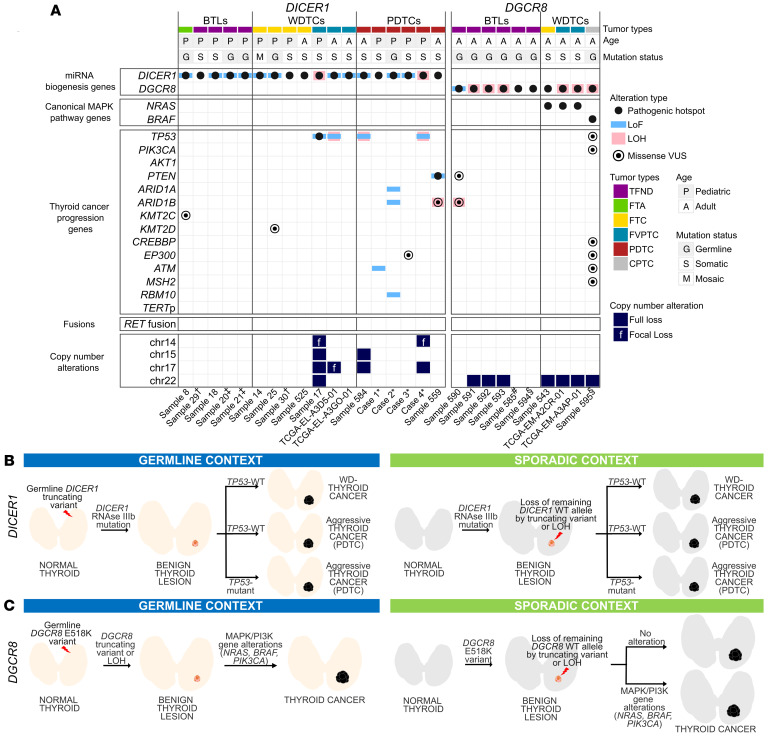
Landscape of *DICER1*- and *DGCR8*-mutated thyroid lesions. (**A**) Molecular landscape of *DICER1*- and *DGCR8*-altered thyroid lesions showing MAPK pathway– or thyroid cancer progression–associated changes and recurrent copy number changes. Benign thyroid lesions (BTLs) harbored *DICER1* or *DGCR8* changes. In well-differentiated thyroid cancers (WDTCs) and poorly differentiated thyroid carcinomas (PDTCs), *DICER1* mutations were mutually exclusive from MAPK gene changes, occasionally co-occurring with *TP53* mutations. *DGCR8*-altered cancers co-occurred with MAPK pathway mutations. Both *DICER1*- and *DGCR8*-mutated thyroid cancers lacked *TERT* promoter changes as well as the most prevalent *RET* fusions (*RET-PTC1* and *RET-PTC3*). All FVPTCs are invasive encapsulated FVPTCs (IEFVPTC) with the exception of TCGA-EL-A3D5-01, which showed follicular-patterned areas, but could not be classified as IEFVPTC or infiltrative FVPTC due to limited histological material. LoF, loss-of-function; LOH, loss of heterozygosity; VUS, variant of uncertain significance, TFND, follicular nodular disease; FTA, follicular adenoma; FTC, follicular thyroid cancer; FVPTC, follicular variant of papillary thyroid cancer; CPTC, classic papillary thyroid cancer. ‡, †, and § show sets of 2 different lesions from a single patient; ^#^TFND with incidental microPTC; *Case IDs from Chernock et al. (12). (**B**) In the germline *DICER1* context, a truncating variant is followed by a somatic RNase IIIb hotspot mutation, leading to BTL formation. In sporadic cases, a somatic RNase IIIb hotspot occurs first, followed by loss of the WT allele (truncating or LOH), sufficient for BTL development. In both contexts, BTLs progress to cancer in the absence of other changes and may progress to more aggressive cancer types (PDTC) via (a) *TP53* mutation or (b) without further changes. (**C**) In the germline *DGCR8* context, a missense c.1552G>A, p.E518K hotspot mutation is followed by LOH or truncation of the WT allele, forming a BTL. This BTL may acquire MAPK (*BRAF*, *RAS*) or PI3K (*PIK3CA*) mutations for malignant transformation. In sporadic cases, the same sequence of *DGCR8* events occurs, followed by MAPK/PI3K changes during progression to cancer.

Thirteen out of the 18 (72.2%) *DICER1*-mutated cases followed the 2-hit mutational pattern of *DICER1* (a *DICER1* RNase IIIb hotspot mutation and a LoF variant or LOH at the *DICER1* locus). In all instances, *DICER1* changes were mutually exclusive from canonical MAPK gene changes, *RET* fusions, or *TERT* promoter changes (Figure 1A). While the 5 *DICER1*-benign lesions harbored mutations exclusively in *DICER1*, some cancers (4 out of 13, 30.7%) additionally harbored *TP53* changes (Figure 1, A and B). Of these 4 (2 PDTCs, 1 FVPTC, and 1 invasive encapsulated FVPTC [IEFVPTC]), 3 presented aggressive features. Other variants in known thyroid cancer progression genes (*PIK3CA*, *PTEN*, *ARID1A*, *ARID1B*, *KMT2C*, *KMT2D*, *EP300*, *ATM*, and *RBM10*) were also observed in individual thyroid cancers (Figure 1A). Mutational co-occurrence with *TP53* was confirmed by analyzing the GENIE cohort (v17.1) that contained 35 *DICER1*-hotspot-mutated thyroid tumors (28 primary tumors and 7 metastases) with *TP53* changes found in 2 out of 9 primary PDTCs and 2 out of 4 PDTC metastases. In contrast with our IEFVPTC (sample 17), no *TP53* change was found in the 22 well-differentiated thyroid cancers (19 primary tumors and 3 metastases), suggesting rather an association between *TP53* changes and more aggressive thyroid cancers.

All 10 *DGCR8* cases harbored the c.1552G>A, p.E518K hotspot variant, which was germline derived in 7 tumors (from 6 patients). All germline carriers developed a TFND; 1 carrier also developed a CPTC and another was found to have a micropapillary thyroid carcinoma (microPTC) within the TFND. In total, 7 out of 10 lesions lost the wild-type (WT) *DGCR8* allele: 6 through LOH or full chromosome 22 deletion, 1 through a truncating *DGCR8* variant, and another showed a low-VAF E518K variant with a copy number alteration (CNA) profile consistent with 22q LOH (Figure 1A and Supplemental Figure 2). In contrast with the *DICER1* cancers, 4 out of 4 (100%) *DGCR8*-associated malignant lesions also harbored canonical thyroid cancer gene changes (*NRAS* hotspot mutations in the FTC and FVPTCs, and a *BRAF* V600E mutation in a CPTC), demonstrating the co-occurence of *DGCR8* and MAPK gene changes (Figure 1, A–C). The *DGCR8*-benign lesions harbored *DGCR8* mutations exclusively, highlighting a progression mechanism involving RAS pathway activation (Figure 1, A and C).

Both *DICER1*- and *DGCR8*-mutant samples exhibited a quiet genome with some recurrent CNAs, including full loss of chromosomes 17 and 22 in *DICER1* thyroid cancers and in *DGCR8* thyroid lesions, respectively (Figure 1A).

### Impact of DICER1 and DGCR8 mutations on miRNA biogenesis.

**Figure 2:**
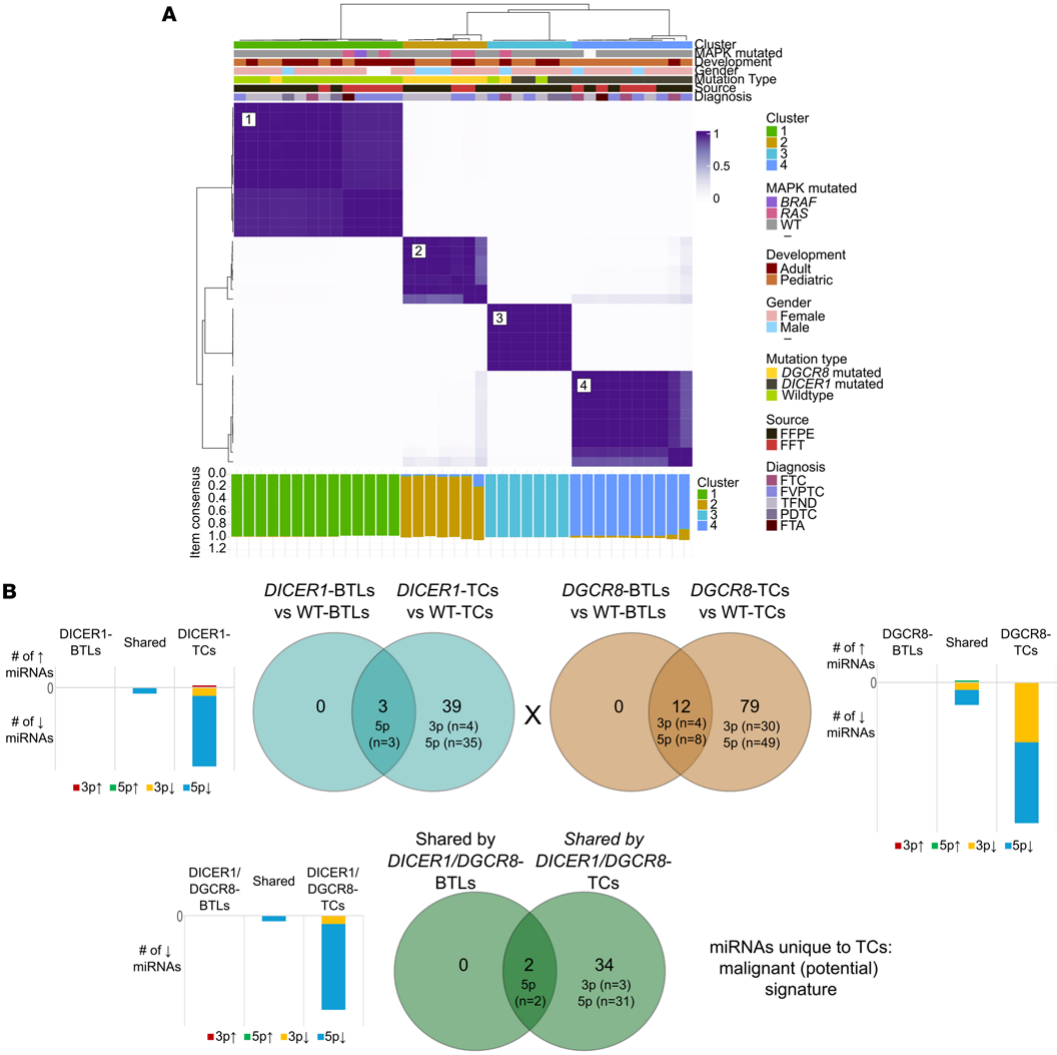
miRNA profiles of *DICER1*- and *DGCR8*-mutated thyroid lesions. (**A**) Unsupervised consensus clustering (1,000 repetitions) of miRNAs in 38 BTLs and malignant thyroid lesions identified: a WT group (cluster 1, *n* = 14) and a *DICER1*-/*DGCR8*-mutant group (*n* = 24), further subdivided into 3 subclusters: (a) a *DGCR8*-mutant group (cluster 2, *n* = 7), (b) a *DICER1*/*DGCR8/*WT mixed group (cluster 3, *n* = 7), and (c) a *DICER1*-mutant group (cluster 4, *n* = 10). The clusters were associated with mutation status and independent of histological diagnosis (benign vs. malignant). Of note, 1 *DGCR8*-mutated TFND clustered with the WT group and 2 WT samples clustered within the *DICER1*/*DGCR8/*WT mixed group. Consensus matrix using the most variable miRNAs with *k* set to 4 (elbow method). (**B**) All differentially expressed miRNAs (DEMs) in the *DICER1* and *DGCR8* BTLs (*n* = 3 and *n* = 12 DEMs, respectively) versus their respective malignant counterparts were persistently downregulated in the thyroid cancers (TCs) that harbored other DEMs, suggesting a linear model of progression. Two DEMs were shared by both *DICER1*- and *DGCR8*-mutated BTLs and TCs, while 34 DEMs were common to *DICER1*- and *DGCR8*-mutated TCs. No DEM was unique to *DICER1*-/*DGCR8*-mutant BTLs. Bar charts show the number of upregulated/downregulated 5p and 3p miRNAs.

To assess the impact of *DICER1* and *DGCR8* mutations on miRNA biogenesis in the thyroid, we profiled the miRNA repertoire of *DICER1*- and *DGCR8*-mutated thyroid lesions compared to WT tumors. Unsupervised consensus clustering of miRNAs from 38 tumors (14 *DICER1*-mutated, 9 *DGCR8*-mutated, and 15 *DICER1-*/*DGCR8*-WT) identified 2 main clusters, including a WT group (cluster 1) and a *DICER1*-/*DGCR8*-mutant group (Figure 2A). The WT group comprised WT samples and 1 *DGCR8*-mutated TFND. The mutant group was further subdivided into 3 subclusters: (a) a *DGCR8*-mutant group (cluster 2), (b) a *DICER1*/*DGCR8/*WT mixed group (cluster 3), and (c) a *DICER1*-mutant group (cluster 4). The clusters were associated with mutation status and were independent of histology (Figure 2A).

### miRNA profiling of benign and malignant lesions with impaired miRNA processing.

Also, we defined the benign versus malignant profiles unique to each driver against each set of WT counterparts. In both entities, the differentially expressed miRNA (DEM) profiles confirmed that changes in miRNA processing leading to nodule development persist through the carcinoma process. We identified 3 DEMs (miR-30a-5p, miR-218-5p, and miR-324-5p), all of which were downregulated in *DICER1*-mutated BTLs compared with WT BTLs (Supplemental Table 4). These same 3 miRNAs and 38 extra downregulated DEMs and 1 upregulated DEM distinguished the cancer status (Figure 2B and Supplemental Table 5), showing an enrichment in certain miRNA families, including let-7 (the family with the most DEMs), miR-30, miR-135, miR-26, miR-221, miR-10, miR-15, and miR-181 (Supplemental Table 6). Following the same trend, between *DGCR8*-mutated and WT BTLs, we identified 12 miRNAs to be differentially expressed (Figure 2B and Supplemental Table 7), belonging to the miR-8, miR-30, and miR-135 families. In addition, 79 miRNAs were found to characterize the *DGCR8* cancer status (Figure 2B and Supplemental Table 8), with an enrichment in several miRNA families (Supplemental Table 9). In all *DICER1*-mutant thyroid lesions (which were all RNase IIIb mutants), we observed a significant reduction in expression of 5p miRNAs, while a reduced expression of both 3p and 5p mature miRNAs was predominant in *DGCR8*-mutant cases, as expected according to previous studies in other tumor types (13, 15, 21, 27–36) (Figure 2B).

### Shared miRNA defects in DICER1/DGCR8 lesions suggest linear thyroid tumor progression.

Owing to the 2 genes’ shared involvement in miRNA biogenesis, we hypothesized that the common 5p miRNA deregulation observed in mutants of both genes is the key event in priming the cell context for the initiation of TFND. Two miRNAs (miR-218-5p and miR-30a-5p) were differentially expressed in both benign and malignant lesions and 34 additional DEMs were unique to mutant malignant thyroid lesions (Figure 2B), supporting an incremental miRNAome deregulation underlying a linear model of carcinoma progression. All 36 DEMs were downregulated, with 92% being 5p mature miRNAs (Figure 2B). Fifteen DEMs were selected for validations by quantitative PCR (Supplemental Figure 3 and Supplemental Table 10).

### Spatially resolved transcriptomic profiling of benign nodules and malignant thyroid cancers with DICER1 or DGCR8 changes.

**Figure 3:**
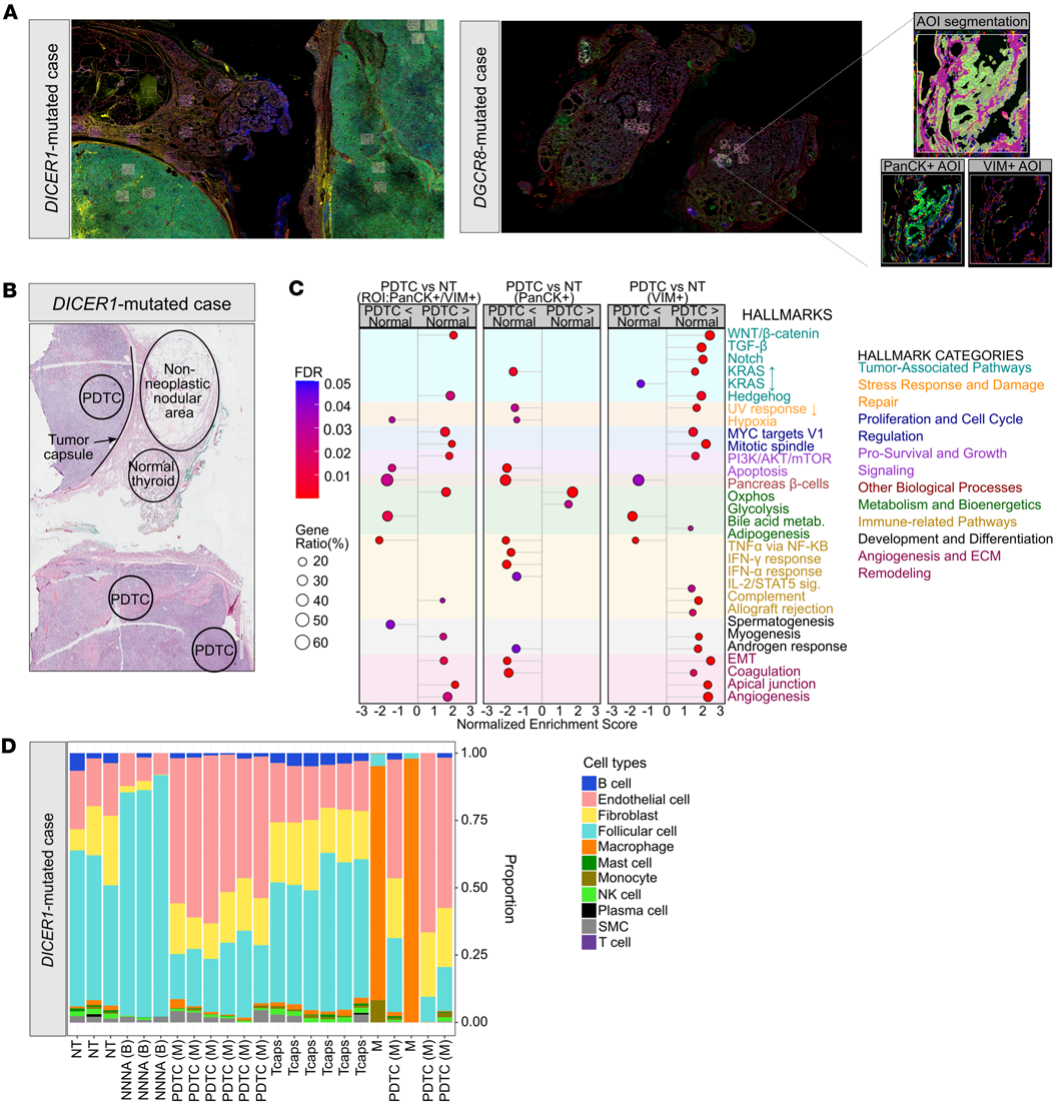
Transcriptomic profiles of a *DICER1* thyroid lesion. (**A**) Immunofluorescent staining for pan-cytokeratin (PanCK) (green) and vimentin (VIM) (red) of a sporadic *DICER1* case and a germline *DGCR8* case. Enclosed areas represent selected regions of interest (ROIs). A minimum of 3 ROIs per histological regions was selected. Inset contains a representative image of the area of illumination (AOI) segmentation for PanCK and VIM. Original magnification, ×20 and ×200 (insets). (**B**) H&E staining of the *DICER1* case showing the selected histological regions (normal thyroid tissue, non-neoplastic nodular area), poorly differentiated thyroid cancer, and the tumor capsule). Original magnification, ×20. (**C**) Dot plot of the significantly altered MSigDB hallmarks of cancer pathways in the poorly differentiated thyroid cancer (PDTC) compared with normal thyroid tissue (NT) at the ROI, PanCK^+^, and VIM^+^ levels. (**D**) Cellular deconvolution of the VIM^+^ component showing an enrichment in endothelial cells in the PDTC. NNNA (B), non-neoplastic nodular area (benign); PDTC (M), poorly differentiated thyroid cancer (malignant); Tcaps, tumor capsule.

We subsequently interrogated the transcriptomic differences between BTLs and malignant thyroid lesions at the spatial level in 2 different tumors individually (1 *DICER1* sample [patient 1] and 1 *DGCR8* sample [patient 2]) by interrogating (a) the follicular cell population expressing pan-cytokeratin (PanCK) and (b) vimentin-expressing (VIM-expressing) cells as a surrogate for enrichment of stromal populations (Figure 3A). We analyzed 4 histological components in patient 1: (a) normal thyroid, (b) a nonmutated non-neoplastic nodular area, (c) a *DICER1*-mutated PDTC, and (d) a capsular region between the PDTC and the normal thyroid tissue and non-neoplastic nodular area (Figure 3B). Gene set enrichment analysis (GSEA) revealed activation of the PI3K/AKT/mTOR pathway when comparing the PDTC with normal thyroid tissue at the region of interest (ROI) level (PanCK^+^VIM^+^) despite the lack of a mutation in *RAS* or PI3K/AKT/mTOR pathway genes (Figure 3C). In the epithelial component (PanCK^+^VIM^–^) of the PDTC, upregulation of oxidative phosphorylation (Figure 3C) and genes related to WNT signaling (Supplemental Figure 4) was observed. In the VIM-expressing cell population (PanCK^–^VIM^+^), we noted activation of cancer proliferation and progression signaling (PI3K/AKT/mTOR, KRAS, and WNT/β-catenin signaling), upregulation of developmental and stem cell pathways (Notch and Hedgehog signaling), involvement of metastasis and invasion processes (epithelial-mesenchymal transition [EMT] and TGF-β signaling), and upregulation of angiogenesis (Figure 3C). The angiogenic upregulation was further reflected by the larger proportion of endothelial cells in the PDTC compared with normal thyroid and the non-neoplastic nodular area, which was inferred from the deconvolution of the VIM^+^ cell populations (Figure 3D). Accordingly, the PDTC’s VIM^+^ component showed significant upregulation of genes involved in extracellular matrix (ECM) formation, angiogenic development, and endothelial function, thus supporting a strong remodeling of the ECM. Of note, the following ECM-related (*COL3A1*, *COL4A1*, *COL4A2*, *FN1*, and *NID1*) and angiogenesis-related (*CDH5*, *FLT1*, *HSPG2*, *KDR*, *PLXND1*, and *SPARC*) genes were direct targets of DEMs seen in the *DICER1* cancers (Supplemental Figure 5).

**Figure 4:**
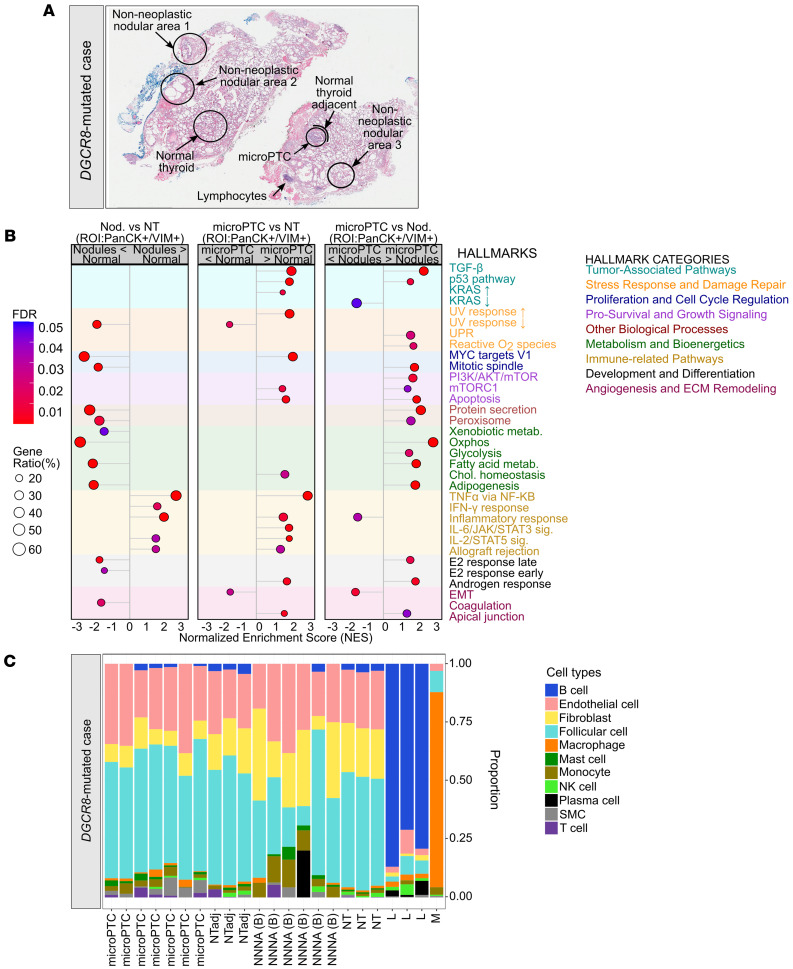
Transcriptomic profiles of *DGCR8*-mutated thyroid lesions. (**A**) H&E staining of the *DGCR8* case showing the selected histological regions: normal thyroid tissue (NT), 3 non-neoplastic nodular areas (benign) [NNNA (B)], a micropapillary thyroid cancer (microPTC), and NT adjacent to the microPTC (NTadj). Original magnification, ×20. (**B**) Dot plot of the significantly altered MSigDB hallmarks of cancer pathways in the NNNAs and the microPTC at the region of interest (ROI) level. (**C**) Cellular deconvolution of the VIM^+^ component showing an enrichment in immune cells. M, macrophage; L, lymphocytes.

Patient 2 harbored a germline *DGCR8* p.E518K mutation and the sample consisted of 4 histological components: (a) normal thyroid, (b) non-neoplastic nodular areas, (c) a microPTC, and (d) normal adjacent thyroid (Figure 4A). Additionally, we captured VIM^+^ colloidal macrophages and CD45^+^ lymphocytes (Figure 4A). In contrast with the *DICER1* sporadic case, we observed gradual changes in the transcriptomic profile from the normal thyroid to the benign non-neoplastic nodular areas to the microPTC at both the epithelial (PanCK^+^VIM^–^) and the stromal (PanCK^–^VIM^+^) levels. We hypothesized that these gradual changes reflected a germline E518K effect and not a tumor-adjacent effect, as no differences were observed between the distal normal thyroid tissue and the normal thyroid adjacent to the microPTC (data not shown). The microPTC was characterized by a remarkable upregulation of KRAS signaling and PI3K/AKT/mTOR signaling compared with the non-neoplastic nodular areas as well as by upregulation of metabolic reprogramming (oxidative phosphorylation, reactive oxygen species, and peroxisome) and of MYC targets (Figure 4B). Pathway analysis between the 3 main histological components (normal tissue, non-neoplastic nodular areas, and microPTC) uncovered a common immune pathway involvement (activation of TNF-α via NF-κB signaling, IL-6/JAK/STAT3 signaling, IL-2/STAT5 signaling, and inflammatory response) in both the non-neoplastic nodular areas and the microPTC compared with the normal tissue. The microPTC showed additional involvement of TGF-β and p53 signaling (Figure 4B). In line with these results, we observed specific upregulation of immune-related genes (Supplemental Figure 6, A and B). The *DMKN* gene was the top upregulated gene common to both nodular areas and microPTC at the ROI, the PanCK^+^, and the VIM^+^ levels and is a direct target of miR-93-5p, which was differentially expressed in the *DGCR8* tumors.

The immune pathway involvement underlying patient 2’s case was not present in the WT non-neoplastic nodular area from patient 1 and was further supported by the presence of different immune cell populations inferred in the VIM^+^ cells of the *DGCR8* lesions (after cellular deconvolution) (Figure 4C). When comparing the normal tissue harboring the germline E518K mutation to the normal tissue from patient 1, the upregulation in inflammatory response, TGF-β signaling, and EMT processes were already detectable, thus supporting an effect of the germline change in the histologically normal thyroid tissue (Supplemental Figure 7).

### Impact of the miRNA production impairment on the expression landscape of mutant BTLs and malignant thyroid lesions.

We hypothesized that DGCR8 dysfunction promotes the inflammatory response observed in these thyroid tumors; thus, we investigated the transcriptional impact of the miRNA dysregulation in *DGCR8*-mutated cases. Integration of the 12 DEMs common to the *DGCR8* BTLs and the *DGCR8* thyroid cancers with the differentially expressed genes (DEGs) seen in the *DGCR8* non-neoplastic nodular areas and the *DGCR8* microPTC showed statistically significant associations with pathways linked to EMT, metabolic reprogramming (oxidative phosphorylation, reactive oxygen species, and peroxisome), and inflammation (IL-2/STAT5 signaling, IL-6/JAK/STAT3 signaling, IFN-γ response, and allograft rejection) (Supplemental Table 11). These results suggest that the first impact of the *DGCR8* change results in an inflammatory process in the thyroid tissue that ignites cancer progression. Of note, the 79 DEMs unique to the *DGCR8* thyroid cancers and the DEGs observed in the *DGCR8* microPTC compared with *DGCR8* non-neoplastic nodular areas were significantly associated with key thyroid cancer pathways (KRAS signaling, mTORC1 signaling, PI3K/AKT/mTOR signaling, p53 pathway, and TGF-β signaling) (Supplemental Table 12). miRNA-mRNA integration for the *DICER1*-mutated lesion was approximate, owing to unmatched dataset groups (Supplemental Methods and Supplemental Table 13).

### miRNA-impaired thyroid lesions exhibit a distinct methylation profile.

*DICER1* thyroid tumors exhibit a wide range of histological subtypes, but *DICER1*-mutated CPTCs have only been reported in a handful of cases (*n* = 4) (37, 38). Consequently, *DICER1* thyroid tumors have been classified as *RAS*-like (26) despite lacking *RAS* mutations. We explored this premise by interrogating the methylation profiles of *DICER1*- and *DGCR8*-mutated thyroid lesions compared to other subtypes.

**Figure 5:**
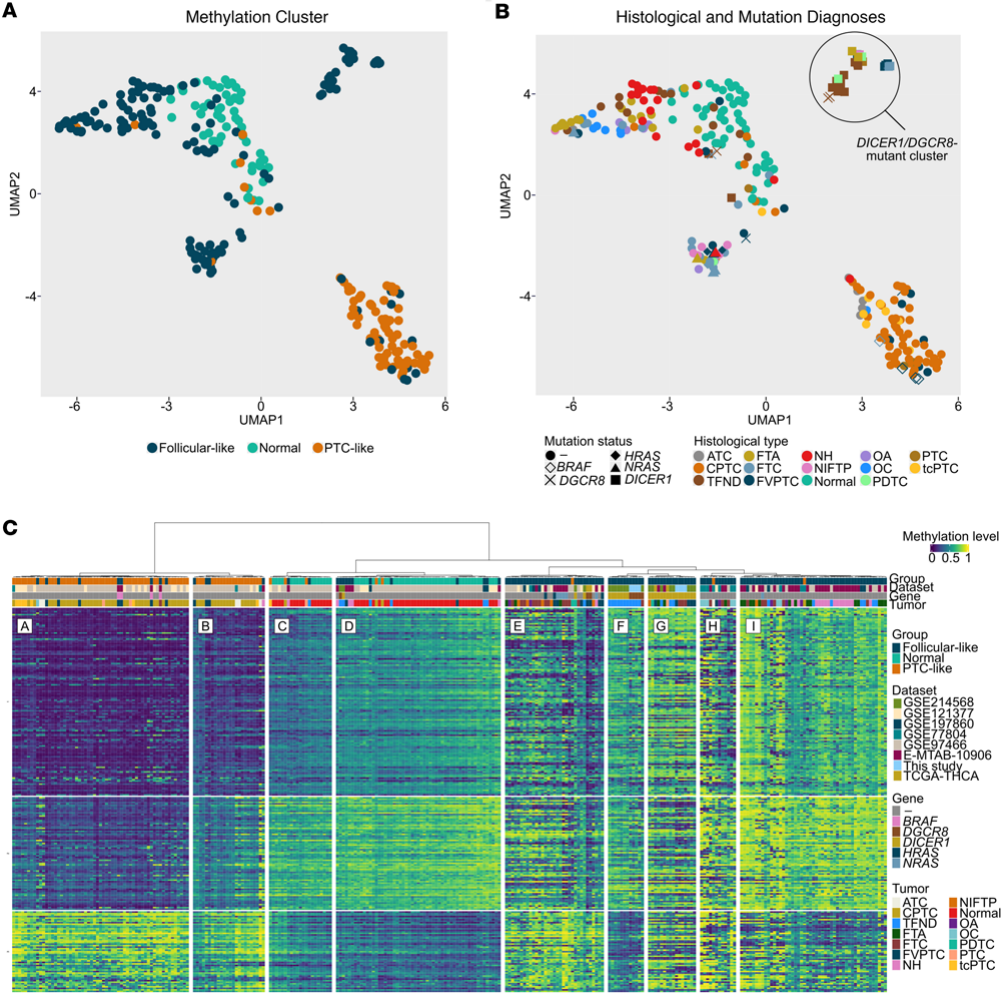
Identification of distinct methylation clusters encompassing *DICER1*- and *DGCR8*-mutated thyroid lesions. Methylation clusters associated with histological diagnosis using the UMAP dimensionality reduction technique. (**A**) Samples are colored according to the methylation clusters observed by Marczyk et al. (39): papillary-like, follicular-like, and normal-like. (**B**) Samples are colored by histological subtype and shapes correspond to gene altered. One follicular-like cluster is made up of *DICER1*- and *DGCR8*-mutated thyroid lesions (*DICER1-*/*DGCR8*-mutated cluster). (**C**) Unsupervised hierarchical clustering (*k* = 9) revealed 3 main clusters (same as those defined by Marczyk et al.; ref. 39). Clusters A and B characterized the papillary-like group and were enriched in classic papillary thyroid cancers (CPTCs), but also contained tall cell PTCs (tcPTCs) and anaplastic thyroid cancers (ATCs). Clusters C and D characterized the normal-like histological group and were enriched in normal thyroid samples. Five clusters characterized the follicular-like group: cluster E, enriched in follicular thyroid cancers (FTCs); clusters F and G, stemming from a shared hierarchical node, were enriched in *DICER1-*/*DGCR8*-benign thyroid lesions and composed exclusively of *DICER1*-mutated samples (enriched in thyroid cancers), respectively; and clusters H and I, also stemming from a common node, were enriched in oncocytic thyroid lesions (OA and OC) and benign thyroid lesions, respectively.

We used 2,279 CpGs from the signature defined by Marczyk et al. (39) to categorize the lesions into follicular-like (*RAS*-associated) and PTC-like (*BRAF*-associated) entities. We benefitted from a publicly available dataset comprising 269 thyroid lesions (35 of which had available molecular data) and integrated it to our methylome data generated from 12 mutant thyroid samples. In total, methylome data from 34 mutant thyroid samples (26 *DICER1* and 8 *DGCR8*) was available. Uniform manifold approximation and projection (UMAP) reduction dimensionality confirmed that thyroid lesions grouped based on histological tumor type, with normal thyroids and follicular-patterned thyroid lesions clustering near each other and away from PTC-like thyroid lesions (Figure 5A). Histological and molecular associations (PTC-like with *BRAF*-V600E and follicular-like with *RAS* mutations) were also confirmed (Figure 5B). This analysis revealed a distinct mutant cluster (*n* = 28) independent of the follicular-like and papillary-like lesions composed solely of *DICER1*-mutated BTLs, *DGCR8*-mutated BTLs, and *DICER1* cancers (Figure 5B).

Unsupervised hierarchical clustering of the 250 most differentially methylated probes supported the presence of the 3 main histological groups defined by Marczyk et al. (39): PTC-like (clusters A and B), normal thyroid (clusters C and D), and follicular-like (clusters E–I). Within the follicular-like group, clusters E, H, and I were enriched in thyroid cancers, oncocytic lesions, and benign thyroid lesions, respectively. Additionally, 32 out of 34 (94%) of *DICER1*-/*DGCR8*-mutated thyroid lesions also belonged to the follicular-like group (Figure 5C). Although the mutant thyroid lesions exhibited a closer hierarchy to follicular-patterned tumors, the unsupervised analysis identified 2 mutant groups stemming from the same hierarchical node corresponding to the *DICER1*/*DGCR8* thyroid lesions highlighted in the UMAP (Figure 5B). On one hand, cluster F was a group strongly enriched in *DICER1* and *DGCR8* BTLs (D1/D8-B hereafter) and cluster G was a group composed solely of *DICER1* lesions, primarily *DICER1* cancers (D1-M hereafter). On the other hand, 2 *DGCR8* cancers (both also harboring a *NRAS* mutation) clustered within a follicular-like cluster containing *RAS*-mutated thyroid cancers (cluster E, Figure 5C). Additionally, 2 mutant TFND samples clustered with the normal thyroid samples. Upon review of the pathological and molecular data, we found that (a) the germline *DGCR8*-mutated TFND sample was highly enriched in normal thyroid tissue and was the only *DGCR8* case retaining a WT copy of *DGCR8*, and (b) the *DICER1* TFND, although harboring a *DICER1* truncating variant, lacked a *DICER1* hotspot mutation. Two *DICER1* cases (1 PDTC and 1 TFND) grouped with the 2 *DGCR8* cancers, found in cluster E, which was enriched in *RAS* tumors. Altogether, these results indicate that *DICER1/DGCR8* lesions share molecular features with other follicular-derived tumors but display a particular epi-signature that can showcase *DICER1* cancers.

### DICER1- and DGCR8-mutated thyroid lesions are characterized by hypomethylation of miRNA-encoding genes.

Differentiated papillary thyroid tumors are characterized by a general hypomethylation, while follicular-like thyroid tumors exhibit a higher proportion of hypermethylated probes compared with normal thyroid tissue (39–41). The miRNA-impaired tumors, although histologically mainly follicular-patterned and clustering with the follicular-like tumors, exhibited a higher proportion of hypomethylated CpGs (located in the open sea), thus behaving rather similarly to papillary thyroid cancers (Table 2 and Supplemental Figure 8).

We then interrogated how the miRNA-impaired tumors (clusters F and G) differed from the rest. Compared with non–*DICER1-*/non–*DGCR8*-mutated follicular-like thyroid lesions (clusters E, H, and I), the miRNA-impaired lesions (clusters F for D1/D8-B and G for D1-M) had 345 and 436 differentially methylated genes (DMGs), respectively (Supplemental Tables 14 and 15). GSEA showed a common significant enrichment of hypomethylated genes corresponding to Gene Ontology (GO) processes related to miRNA-mediated gene-silencing mechanisms (Supplemental Tables 16 and 17). This finding was further validated by an enrichment of individual miRNA genes within the individual hypomethylated genes (Supplemental Tables 14 and 15). Hypomethylation affected mostly the miRNA gene promoters. Likewise, enrichment in GO terms related to miRNA-mediated gene silencing was observed in miRNA-mutated cases compared with papillary-like lesions, supporting this hypomethylation signature as a feature of miRNA-mutated samples (Supplemental Tables 18 and 19).

### Evolving methylation profile of DICER1 thyroid cancers compared to mutant benign lesions.

**Figure 6:**
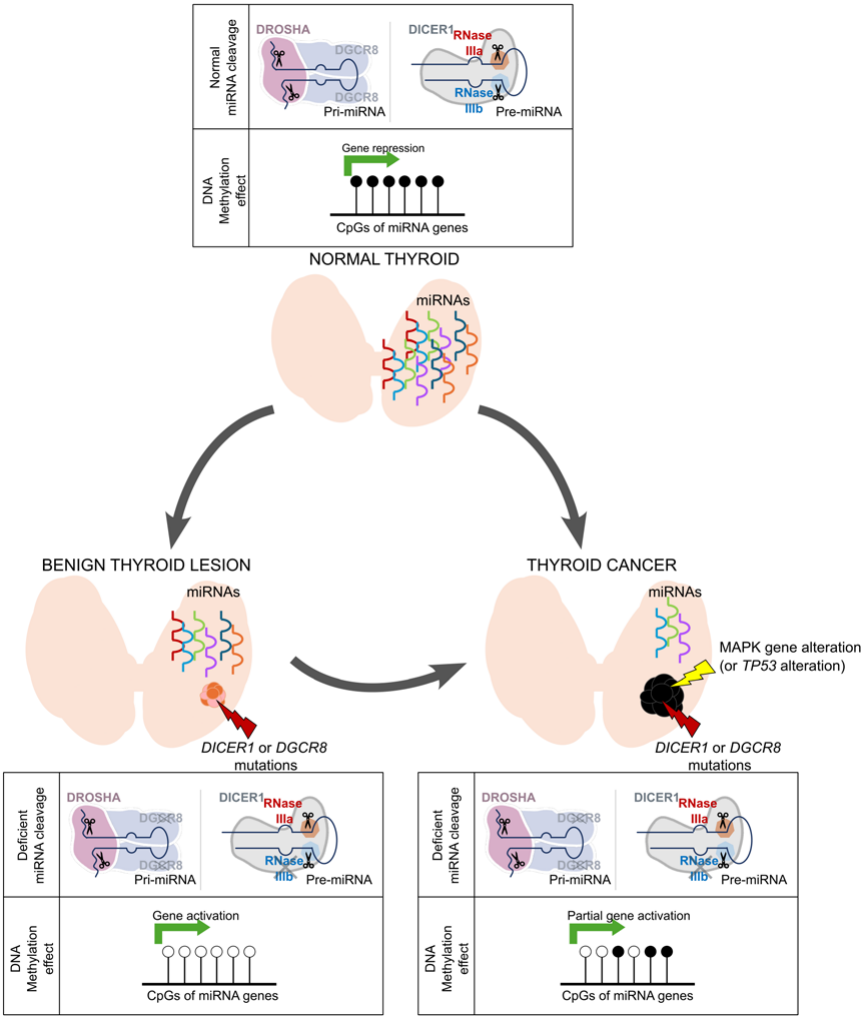
Scheme of epigenetic miRNA compensation mechanism in benign and malignant mutant lesions. We propose a compensatory mechanism involving regulation of miRNA genes at the epigenetic level where both benign and malignant mutant thyroid lesions activate gene expression of miRNA genes in response to lack of miRNA production, in contrast with gene repression of miRNA genes by DNA methylation in the context of normal miRNA production in normal thyroid. This compensatory mechanism was more active in the mutant benign thyroid lesions, supporting successful rescue of some miRNA processing exemplified by a less prominent loss of miRNAs. In the mutant thyroid cancers, we observed a decreased activity of this compensatory mechanism, supported by their greater loss in miRNAs.

*DICER1* benign and malignant lesions had subtle differences in their mutational profiles, with *DICER1*/*DGCR8* genes equally mutated in both; however, deficiencies in miRNA production were severely pronounced in the cancers. Since *DICER1-*/*DGCR8*-mutant BTLs exhibited a lower global DNA methylation level than cancers (at the general level and when focusing on differentially methylation probes [DMPs]) (Table 2 and Supplemental Figure 9), we interrogated methylation profiles possibly associated with carcinoma progression. We identified 322 DMGs between the mutant malignant (D1-M) and benign (D1/D8-B) groups (Supplemental Table 20). Although *DICER1-*/*DGCR8*-mutant lesions were characterized by hypomethylation of miRNA-mediated gene-silencing processes, these processes were altered to a lesser extent in *DICER1*-driven cancers (Figure 6 and Supplemental Table 21). In fact, when looking at individual miRNA genes, benign lesions showed twice the number of hypomethylated miRNA genes compared with malignant lesions (40 vs. 24 DMGs compared with normal thyroid, respectively) (Figure 6 and Supplemental Tables 22 and 23). Five tumor suppressor miRNA genes (*MIR517A*, *MIR519D*, *MIR1266*, *MIR548C*, and *MIR711*) were specifically hypermethylated in the mutant cancers compared with the benign forms; of note, *MIR1266* and *MIR548C* are associated with tumor growth and metastasis (42, 43).

As a feature, *DICER1* cancers (D1-M) showed an enrichment of hypermethylated genes in biological processes related to immune response, cell signaling, and migration, which might underlie a putative silencing mechanism leading to immune evasion and impaired tissue remodeling. The gene enrichment processes with a marked hypomethylation involved mechanisms of adaptation to cellular stress such as enhanced metabolic pathways and DNA repair, proper to cancer processes (Supplemental Table 15). Looking at specific genes, we noted that *DICER1*-mutant thyroid cancers displayed differential hypomethylation in the promoters of *MIRLET7G* (a top DEM in the cancers), *KRT19*, *CTPBP1*, *CXXC5*, *PRAME*, *THRSP*, and *FERMT1*, as well as hypermethylation in the promoters of *ETS1*, *TGFRB1*, *EYA1*, *FGF2*, and *SERPINF1*, all of which are involved in thyroid cancer progression (21, 44–60). We hypothesized that these methylation changes might result from loss of miRNA regulation of methylation enzymes. Analysis of cancer-specific DEMs identified 24 targeting *DNMT1/3A/3B* and *TET1*/*2/3* genes. Evaluation of the spatial transcriptomic data revealed significant upregulation of *DNMT3B* and lower expression of the eraser *TET2* in the epithelial component of the *DICER1* PDTC compared with the corresponding normal tissue (*P* = 0.0121 and *P* = 0.0242, respectively).

## Discussion

The tumor susceptibility syndromes caused by GPVs in *DICER1* and *DGCR8* microprocessors are rare entities that predispose to the development of early-onset benign and malignant thyroid tumors. Somatic changes in these genes are also responsible for a proportion of thyroid sporadic tumors yet the routes to malignant progression, driven by defective miRNA production, in these entities is unclear. In the present study, we depict a linear evolution model toward malignant transformation marked by accumulation of miRNA deficiencies paired with a characteristic epigenomic signature of *DICER1* and *DGCR8* lesions.

While *DICER1* downregulation has been described as a risk factor for thyroid cancer progression and aggressiveness (61), thyroid tumors in patients with DRTP present a very specific mutational pattern, not tolerating a complete loss of the *DICER1* gene (62). A similar mutational scenario occurs in *DGCR8* thyroid tumors resulting in hemizygous-expressing tumors with a functionally impaired DICER1 or DGCR8 protein. By analyzing a compendium series of miRNA-impaired thyroid tumors, we define 2 genomically distinct models of progression for *DICER1* and *DGCR8* thyroid lesions (Figure 1, B and C). For *DICER1*-mutated thyroid lesions, a mutually exclusive relationship between *DICER1* and MAPK gene changes is observed, confirming our findings in nodules with indeterminate cytology (23) as well as in other reports of *DICER1*-mutant tumors (12, 20–22, 63). The transcriptomic profile of a *DICER1* PDTC showing activation in main thyroid cancer pathways further supports a mechanism wherein *DICER1* mutations alone are sufficient to promote the development of BTLs as well as invasive thyroid cancers (Figure 1B). In fact, some *DICER1* thyroid cancers carry only a heterozygous hotspot mutation (12, 21, 23, 25, 64, 65), suggesting it may be sufficient for tumorigenesis and pointing to possible nongenomic factors involved in cancer progression, although other undetected *DICER1* variants (66–72) cannot always be excluded.

*TERT* promoter mutations as well as *TP53* changes are classically associated with tumor progression and bad prognosis (64, 73–79). In *DICER1* tumors, while *TERT* changes were absent, *TP53* was the most frequently mutated gene in *DICER1* PDTCs. This co-occurrence was present even in a pediatric *DICER1* DTC despite *TP53* rarely being altered in pediatric thyroid tumors. Interrogation of the GENIE cohort supported the idea that a *DICER1*- and *TP53*-mutated tumor harbors potential for further dedifferentiation along with the *TP53*-associated poor prognosis, underscoring the importance of *TP53* testing in *DICER1* thyroid tumors of all ages.

Progression from a *DGCR8*-mutated benign tumor to DTC required an additional somatic MAPK or PI3K/AKT/mTOR pathway change, with 100% of our DTCs harboring MAPK pathway changes (Supplemental Table 2). While *DGCR8* follicular-patterned thyroid cancers harbored *NRAS* mutations, a germline *DGCR8* E518K carrier (14) developed a TFND and a subsequent CPTC with a *BRAF* V600E mutation, consistent with known associations of *RAS* and *BRAF* mutations in follicular-patterned thyroid cancers and CPTCs, respectively (80–82) (Supplemental Table 2). This suggests that the DGCR8 deficiency primes the thyroid cell and its microenvironment to develop a benign nodule, but an accompanying MAPK gene mutation boosts cancer progression and determines the histological subtype. Conversely, *RAS* oncogenic mutations are not sufficient to drive thyroid cancer (83); thus, a thyroid cell with an underlying *DGCR8* deficiency would provide an ideal setting for *RAS*-driven tumorigenesis, even more so since a 22q loss event is highly associated with *DGCR8*-E518K mutants and is an important cofactor in *RAS*-driven cancers (84). This effect is reinforced by reports of other *DGCR8*-mutated FTCs harboring *RAS* and *PIK3CA* mutations (15, 16).

Keeping with DICER1’s master role as an miRNA processor, *DICER1*-mutant tumors have shown to exhibit 5p miRNA downregulation as well as upregulation of 3p* (passenger strands) miRNAs (21, 29, 30, 32, 85); however, passenger strands are reported to be highly unstable (86). In the case of *DGCR8*-E518K tumors, this 5p miRNA downregulation is paired with 3p miRNA downregulation, reflecting the aberrant processing of the pre-miRNA leading to subsequent loss of the mature miRNAs stemming from it (13). Shared deregulated miRNAs between the BTLs and malignant thyroid lesions in *DICER1-*/*DGCR8*-mutated thyroid lesions supported a linear model of progression parallel to the one in other follicular thyroid tumors (87–89). In this scenario, 5p miRNAs downregulated in the BTLs would represent a tumor initiation defect active in benign TFNDs while the thyroid cancers accumulate additional deregulated miRNAs that feature the cancer progression. miR-218-5p and miR-30a-5p, downregulated in BTLs, were significantly associated with G_2_/M checkpoint and KRAS signaling (concordant with FTAs harboring *RAS* mutations) (64, 90, 91), yet their effects on EMT and inflammatory processes might underlie a remodeling of the tumor cells and its microenvironment susceptible to progression.

Tracing their route to malignant transformation, we identified a deficiency in miRNAs involved in thyroid carcinogenesis, including let-7 family members (that target RAS signaling effectors), miR-135 (15, 21), and many others known to regulate PI3K/AKT/mTOR signaling (61) (e.g. miR-221, miR-222, miR-99a, miR-451a, and miR-21), mimicking transcriptomic disturbances required for progression in *RAS*-mutated follicular tumors. This drastic miRNA processing impairment impacts main thyroid cancer pathways (MAPK, PI3K/AKT/mTOR, and TGF-β pathways, among others), leading to a general loss of repressive regulation. Conversely, oncomiRs typically upregulated in thyroid cancers do not escape this deficiency. Consequently, thyroid cancers with DICER1 or DGCR8 deficiency show downregulation of PI3K pathway regulators (61) (including miR-146, miR-21, miR-34a, miR-221, miR-29b, and miR-23b), as well as miR-222, miR-191, and miR-324, previously proposed as biomarkers for invasion and metastasis (92–95). This downregulation may explain the low metastatic potential of these tumors and stresses how these miRNAs are ineffective malignancy biomarkers to detect *DICER1-*/*DGCR8*-driven cancers.

The *DICER1* PDTC transcriptome reflected how the global miRNA deregulation caused by a *DICER1* hotspot change holds progression potential affecting several pathways (WNT/β-catenin signaling, EMT, and angiogenesis) (96–100) in the absence of canonical MAPK driver gene mutation, although these preliminary observations need to be validated in other *DICER1* PDTCs. Specifically, the *DGCR8*-mutated case demonstrated a strong involvement of immune processes, a shared feature with undifferentiated thyroid cancers (101–104) that prompted us to investigate defects of the *DGCR8* tumors that might be related to an inflammatory process. EMT dysregulation has previously been associated with high-grade and invasive thyroid neoplasms (105); however, the microPTC showed no such features and the involvement of EMT was detected in the *DGCR8* TFND areas compared with nonmutated TFND and normal tissue. Thus, this dysregulation could rather be attributable to the 3p miRNA losses characteristic of *DGCR8* lesions. In particular, miR-200 and miR-141 belong to the same family, are exclusively downregulated in *DGCR8* tumors, target TGF-β signaling (61), are epigenetically regulated, and play a fundamental role in maintaining the epithelial identity of the cell. In follicular cells of anaplastic thyroid carcinomas (ATCs), loss of this miRNA family promotes a mesenchymal phenotype, marked by increased VIM and decreased E-cadherin expression (44).

The transcriptomic profile shows that heterozygous *DGCR8* E518K mutation primes the tissue for tumorigenesis by affecting the microenvironment, e.g., by promoting EMT and an inflammatory response in the normal tissue that is sustained in both BTLs and malignant thyroid lesions of this patient (findings that should be investigated further in additional *DGCR8*-mutated thyroid lesions). Upon investigation of the other *DGCR8*-mutated cases, their clinical notes revealed lymphocytic thyroiditis in 5 out of 6 *DGCR8* TFNDs and 1 out of 3 *DGCR8* thyroid cancers, which reinforced a role of *DGCR8* malfunction in the initiation of an inflammatory response.

Although miRNA-biogenesis-mutant thyroid lesions are considered similar to *RAS*-like entities, their methylation profile displayed unique features shared by 2 independent mutant groups: a group enriched in *DICER1*-/*DGCR8*-mutated BTLs and a group solely composed of *DICER1* lesions (cluster G). This result implied a particular epi-signature existing in *DICER1* tumors; hence, we investigated the clinical data of the samples in cluster G (Figure 5C). The group comprised a majority of thyroid cancers (*n* = 10), 1 low-risk neoplasm (NIFTP), 2 FTAs predicted to be malignant (106), and 3 TFNDs (including 2 in which the possibility of a follicular neoplasm could not be entirely excluded according to their pathology reports). This pointed to a methylation signature characteristic of *DICER1* malignant tumors that could be exploited in differential diagnosis or to identify prognostic markers in benign lesions.

The global hypomethylation in miRNA-biogenesis-impaired tumors was strongly related to miRNA silencing, especially in the benign lesions (Supplemental Figure 8). Given that canonical miRNA genes are transcribed as mRNAs and subjected to pre- and posttranscriptional regulatory mechanisms (86), this hypomethylation might outline a compensation mechanism in response to reduced miRNA-mediated silencing that is lost through tumor progression.

In addition to the omic profiles presented here, prior studies have explored cytological (107, 108), histological (109–112), and ultrasonographic features (113) characteristic of *DICER1*-related thyroid tumors, proposing ghost-like infarctions as pathognomonic for *DICER1*-related thyroid cancers. Together, integrating these molecular and morphological features may enhance identification of *DICER1*-mutated cases, facilitating early diagnosis of germline *DICER1* carriers and enabling implementation of clinical surveillance strategies for patients and at-risk family members.

### Limitations

Limitations inherent to sample size when working with rare diseases: Direct integration of miRNA and transcriptomic analysis in *DICER1*-mutated cases was not possible since the PDTC case was sporadic. PDTC-associated miRNAs could not be extrapolated since only 2 cases were available. While we did not aim to directly compare the transcriptomic differences between the *DICER1*- and the *DGCR8*-mutated thyroid lesions, we recognize that their differences may reflect the distinct clinical behaviors associated with the 2 histological types analyzed.

### Concluding remarks

Despite the similarities between *DICER1-*/*DGCR8-*mutant BTLs and malignant thyroid lesions with respect to mutational landscape and genomic integrity, a marked increase in the repertoire of miRNA deficiencies characterized the malignant forms independently of the altered gene. Multiomic results suggest that in *DGCR8*-mutated cases, an inflammatory process may set the scene for tumor susceptibility; however, mutational events affecting the MAPK pathway steer tumorigenesis toward transcriptomic and methylomic profiles characteristic of *RAS*-like tumors. In *DICER1* cancers, deficiencies in miRNA processing are sufficient to alter transcriptional programs and disrupt thyroid tissue homeostasis. Hypomethylation of pri-miRNA genes underlies a compensatory mechanism to alleviate the deficiency in miRNA production. The specific pattern of miRNA downregulation and methylation of miRNA genes opens the way to establish malignancy biomarkers in these tumors, with the let-7 family members emerging as strong candidates.

## Methods

### Sex as a biological variable.

Our study included male and female patients. However, 2 inherent biases existed in our cohort: the female-to-male ratio for thyroid lesions is typically 4:1; thus, our cohort was enriched in females and patient selection was based on *DICER1* and *DGCR8* mutation status and due to the rarity of these cases, no consideration was taken to obtain a sex-matched sample cohort.

### Participant details.

We collected 581 benign and malignant thyroid tumors including 63 pediatric and 518 adult samples from 60 (57 unpaired and 3 paired) and 365 (223 unpaired, 133 paired, 6 two-to-one paired, and 3 two-to-two paired samples) individual pediatric and adult patient cases, respectively. The 251 samples of benign histology (14 pediatric, 238 adult) consisted of FTA, oncocytic thyroid adenoma (OA), and TFND. The 330 samples of malignant histology (49 pediatric, 280 adult) consisted of papillary thyroid carcinoma (PTC), FVPTC, FTC, oncocytic thyroid carcinoma (OC), PDTC, and ATC. To increase our cohort of samples with miRNA biogenesis defects, we additionally included 1 *DICER1*-mutated sample (1 PDTC identified clinically at the pathology department of the Jewish General Hospital) and 1 *DGCR8*-mutated sample (1 TFND identified clinically at the endocrinology department of the Hospital for Sick Children [Toronto]), all of which (*n* = 2) have never previously been published. We further included 4 *DICER1*-mutated (4 PDTCs) and 8 in-house *DGCR8*-mutated samples (5 TFNDs, 1 CPTC, and 2 FVPTCs) from previous studies (12–14) (Supplemental Figure 1).

For greater statistical power, data derived from samples of the current study were merged with data from in-house unpublished and published *DICER1-*/*DGCR8*-mutated cases and/or from public databases (Table 1 and Supplemental Figure 1). Table 1 details the provenance of the mutated cases in each experiment.

### WES.

WES was performed on 10 *DICER1*-hotspot-mutated samples (in-house cases and identified in the genotyping phase) and 8 *DGCR8*-hotspot-mutated samples (from the genotyping phase and in-house cases) at the Centro Nacional de Análisis Genómico (CNAG). The *DICER1* samples included 5 benign samples (2 TFNDs from individual patients, 2 TFND samples from the same patient, and 1 FTA), and 5 malignant samples (3 FTCs, 1 FVPTC, and 1 PDTC). The *DGCR8*-mutated samples included 6 benign samples — 5 TFNDs (13, 14) and 1 TFND with a microPTC, and 2 malignant samples — 1 CPTC (14) and 1 FTC. WES library preparation, variant calling, and variant filtering details are found in the Supplemental Methods.

### miRNA profiling experiment.

The miRNA profiling experiment consisted of 38 samples: 12 thyroid cases from a previous miRNA profiling experiment (13) and 26 cases were from an in-house sample cohort. To summarize, the benign sample cohort comprised 16 BTLs (5 WT, 5 *DICER1*-mutated, and 6 *DGCR8*-mutated), while the malignant sample cohort included 9 non–hotspot-mutated tumors (CPTC, *n* = 1; FTC, *n* = 1; FVPTC, *n* = 5; PDTC, *n* = 2), 10 *DICER1*-mutated tumors (FTC, *n* = 3; FVPTC, *n* = 5; PDTC, *n* = 2), and 3 *DGCR8*-mutated tumors (FTC, *n* = 1; FVPTC, *n* = 2).

All samples were analyzed using the NanoString nCounter Human v3 miRNA Expression Assay (NanoString Technologies) according to the manufacturer’s instructions at the Lady Davis Institute’s Molecular Pathology Research Core. Samples from different sources (FFPE or FFT) and with different mutation statuses for *DICER1*/*DGCR8* were evenly distributed across the different panel chips performed (version 3a [*n* = 5]; version 3b [*n* = 25]). Details regarding miRNA profiling, quality control, and data normalization are the Supplemental Methods.

### miRNA differential expression analysis.

miRNA data expression was normalized using the trimmed mean of M (TMM) values, and effective library sizes were calculated. miRNAs with 10 or more counts per million (CPM) in more than 70% of the samples were retained. Subsequently, differential expression analysis was performed using the quasi-likelihood negative binomial generalized log-linear model (GLM) functions provided by the EdgeR package v4.10.16 (https://bioconductor.org/packages/release/bioc/html/edgeR.html), adjusting for experiment batch, chip version, and sample source. Comparisons were made between hotspot-mutated and WT nodules (whether benign or malignant). Additionally, mutant malignant groups were compared to their mutant benign counterparts. Significance for DEMs was defined as an FDR of less than 0.05 and an absolute log_2_(fold change) (|log_2_FC|) of 2 or greater. ConsensusClusterPlus package was used for subgrouping (1,000 resamplings) (114). Target analysis of DEMs was performed using miRnet (https://www.mirnet.ca) through the miRTarBase v9.0 database. miRNA family enrichment was defined by 2 or more differentially expressed miRNAs per family. RT-qPCR validation details are provided in the Supplemental Methods.

### Spatial transcriptomics analysis.

We profiled a total of 51 ROIs in 1 sporadic *DICER1*-mutated case and 1 germline *DGCR8*-mutated case at spatial resolution using the GeoMx platform (NanoString Technologies). We first captured an epithelial component expressing the PanCK marker and aimed to profile the stromal population by capturing VIM-expressing cells. For the *DGCR8*-mutated case, we also aimed to profile the case’s immune profile by capturing CD45-expressing lymphocytes. Differential gene expression between areas of illumination (AOIs) was performed with Limma v3.56.1 (115). Genes were considered differentially expressed with an FDR of 0.05 or less. Functional analyses of DEGs included GO, KEGG, and GSEA via the clusterProfiler v4.9.0.002 (116) package. Terms with an FDR of 0.05 or less were considered significant. Cell deconvolution of each AOI was performed using SpatialDecon v1.10.0 (117). A custom profile matrix was created from publicly available single-cell data (GSE191288), which was processed using Seurat v3 (118) and annotated using the Annotation of Cell Types (119) web server. Additional details pertaining to the spatial transcriptomic profiling sample preparation, ROI selection and capture, data quality control and normalization are in the Supplemental Methods.

### DNA methylation.

DNA methylation profiling was performed using Illumina 450K, EPICv1, and EPICv2 arrays. Data were preprocessed with Noob normalization, and poor-quality, sex chromosome, SNP-containing, and cross-reactive probes were removed. Batch effects related to array and material type were corrected. Global methylation levels and regional patterns were assessed using mean β-values across CpG island–associated and non-island regions. Unsupervised clustering (UMAP and hierarchical) and heatmap visualization were used to identify methylation-based tumor subtypes. DNA methylation analyses were performed using the ChAMP pipeline (FDR < 0.05, |Δβ| > 0.2) to identify differentially methylated positions (DMPs), genes (DMGs), and regions (DMRs), with DMRs called using the ProbeLasso method implemented in ChAMP. See Supplemental Methods for detailed preprocessing, probe filtering, clustering, and enrichment.

### Statistics.

Statistical methods were applied consistently across molecular datasets to identify differentially expressed or methylated features and their biological relevance. For miRNAs, TMM normalization and quasi-likelihood GLMs were used, adjusting for batch effects; significance was defined as an FDR of less than 0.05 and |log_2_FC| of 2 or greater. Spatial transcriptomic data were analyzed with Limma (https://bioconductor.org/packages/release/bioc/html/limma.html), followed by functional enrichment (GO, KEGG, Hallmarks, GSEA) using clusterProfiler. Cell-type deconvolution applied SpatialDecon with a custom single-cell RNA-seq–derived reference. Integration of miRNA-mRNA data employed Fisher’s exact test and FDR correction to assess pathway enrichment using MSigDB gene sets. DNA methylation analyses included identification of DMPs, DMGs, and DMRs using CHAMP (https://bioconductor.org/packages/release/bioc/html/ChAMP.html) and ProbeLasso, with FDR less than 0.05 and |Δβ| greater than 0.2. Dimensionality reduction (UMAP) and hierarchical clustering informed unsupervised subtype classification, and pathway analysis used methylGSA (https://bioconductor.org/packages/release/bioc/html/methylGSA.html) and FGSEA (https://bioconductor.org/packages/release/bioc/html/fgsea.html), with all *P* values adjusted via Benjamini-Hochberg correction.

### Study approval.

Samples were recruited under informed consent for genomic analysis at the recruitment sites. Control samples were recruited anonymized from the Hospital Universitari de Bellvitge biobank. Ethics approval for the collection, use and genomic analysis of the patient samples was obtained from the Hospital Universitari de Bellvitge Ethics Committee (PR108/20) and the Comitè d’Ètica d’Investigació amb medicaments (CEIm) Fundació Sant Joan de Déu (PIC-253-20) and the A.C. Camargo Cancer Center Ethics Committee (CAAE:84725318.4.0000.5432).

### Data availability.

miRNA profiling and DNA methylation data files are deposited at NCBI’s Gene Expression Omnibus (GEO) under GSE301150 and GSE300956. The expression count matrix from the spatial transcriptomic experiment is deposited under GSE301163. The WES datasets generated in this study are available in the European Genome-phenome Archive (https://ega-archive.org/) under study ID EGAS50000001577. Raw data supporting Supplemental Figures 3, 8, and 9 can be found in the Supporting Data Values file.

## Author contributions

ASC performed experiments, analyzed data, generated figures, and wrote the original manuscript draft. C Roca and PMS performed experiments, analyzed data, and generated figures. ED and C Rovira analyzed data. VB and LJLG performed experiments. MP, IRC, and DBDS provided resources and analyzed data. PV, CJ, RDC, GTT, XMG, CVA, JMCT, HS, JDW, WDF, and PCS provided resources. EAL analyzed data and developed methodology. BR contributed to project conceptualization, supervision, funding acquisition, project administration, and wrote and reviewed the manuscript.

## Funding support

- Fundación Científica Asociación Española Contra el Cáncer (AECC) (grant LABAE235269RIVE) to BR.
- Fundación Mutua Madrileña (grant AP173972020) to BR.
- Fundación La Marató de TV3 (grant 202031-10) to BR.
- Agència de Gestió d’Ajuts Universitaris i de Recerca (AGAUR) (grant 2021 SGR 01066) to BR.
- Instituto de Salud Carlos III (ISCIII) and the European Social Fund: Investing in Your Future (grant CP21/00038) to BR.
- “La Caixa” Foundation (ID 100010434) fellowship LCF/BQ/DI21/11860051 to ASC.
- Agencia Estatal de Investigación (grant PID2022-140149OB-I00) to CVA and to the TIROCHUS collection [Rnb code: ISCIII-BIO-2012/000026]).
- ISCIII (grant PI23/00722) to JMCT.
- Fundação de Amparo à Pesquisa do Estado de São Paulo (grants 2018/06269-5 and 2020/00870-9) to GTT.
- Comunidad de Madrid (funding for the iTIRONET Consortium [P2022/BMD7379], of which LJLG, PV, and IRC are members).

## Supplementary Material

Supplemental data

Supporting data values

## Figures and Tables

**Table 1:**
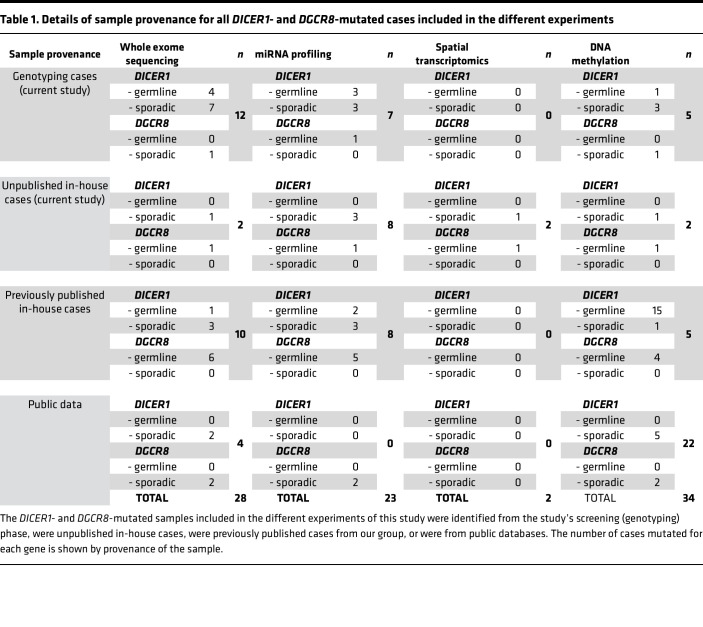
Details of sample provenance for all *DICER1*- and *DGCR8*-mutated cases included in the different experiments

**Table 2:**
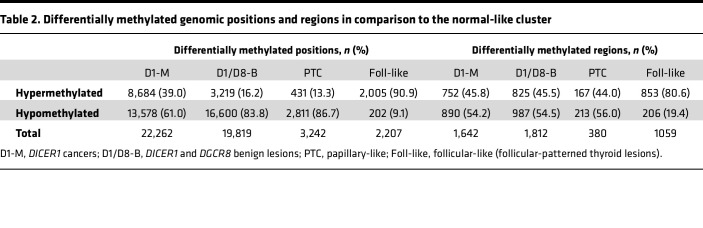
Differentially methylated genomic positions and regions in comparison to the normal-like cluster

## Version 1 02/09/2026

Electronic publication
