# Supplementary material for: Tracing the molecular route to progression in miRNA-biogenesis-defective thyroid lesions
Source: JCI Insight. 2026 Feb 9;11(3):e198338. doi: 10.1172/jci.insight.198338 (PMC12893109; doi:10.1172/jci.insight.198338)
Supplement: Supplemental data [file jciinsight-11-198338-s230.pdf]

SUPPLEMENTAL FIGURES

Figure S1: Sample provenance for all *DICER1*- and *DGCR8*-mutated cases included in the different experiments

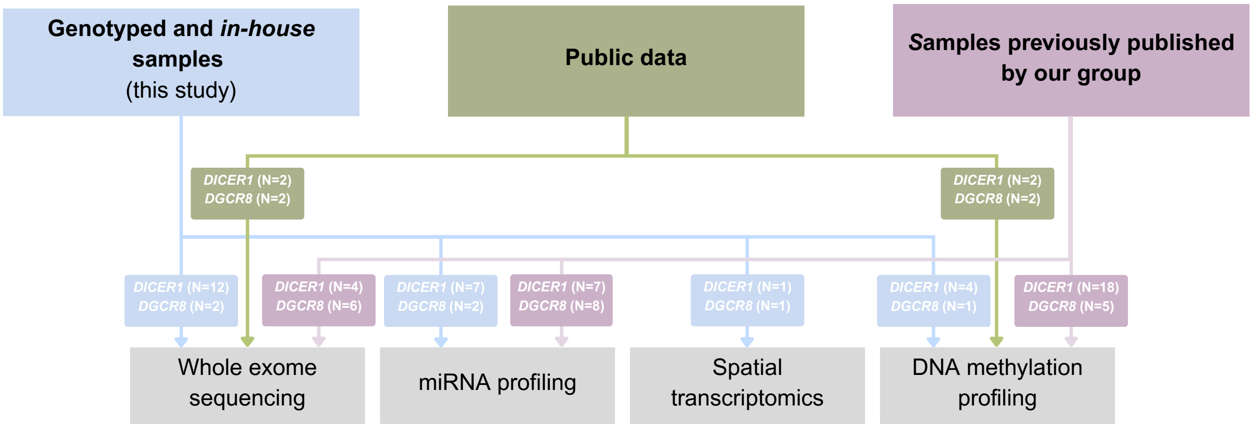

Flowchart showing the number of *DICER1*- and *DGCR8*-mutated samples included in each experiment and their provenance. The blue set includes samples identified by the current study during the genotyping phase together with in-house samples previously identified to be mutated and that have never been published before; the pink set includes a sample set previously published by our group that was further included in the profiling experiments; and the green set includes data stemming from publicly available studies.

Figure S2: Copy number alteration of chromosome 22 of in *DGCR8*-follicular thyroid cancer (sample 543)

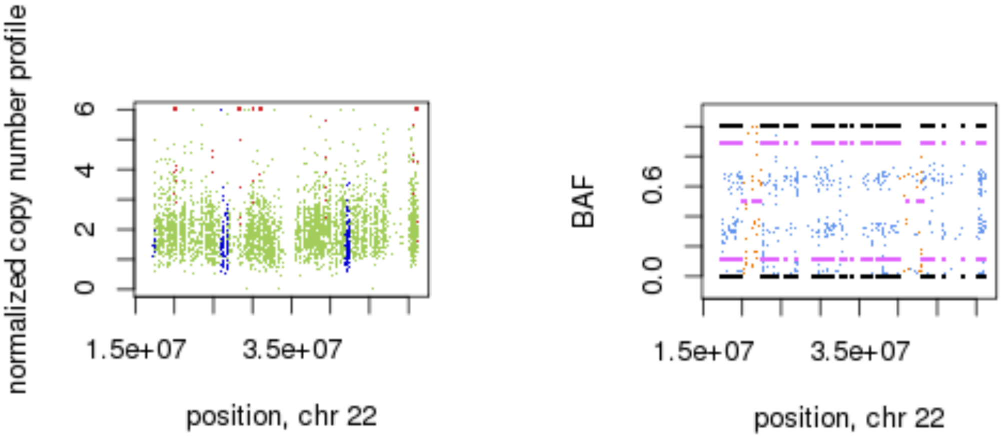

Chromosome 22 copy number and B-allele frequency (BAF) profiles of sample 543 (*DGCR8*-follicular thyroid cancer). Left panel shows chr22 copy number N=2 in the DGCR8-thyroid cancer. Right panel shows predicted BAF around 0.30 and 0.60 in the *DGCR8*-thyroid cancer. Copy number N=2 and estimated BAFs of 0.30 and 0.60 are suggestive of chr22 loss in a clonal population of cells.

Figure S3: miRNA expression validation by quantitative PCR

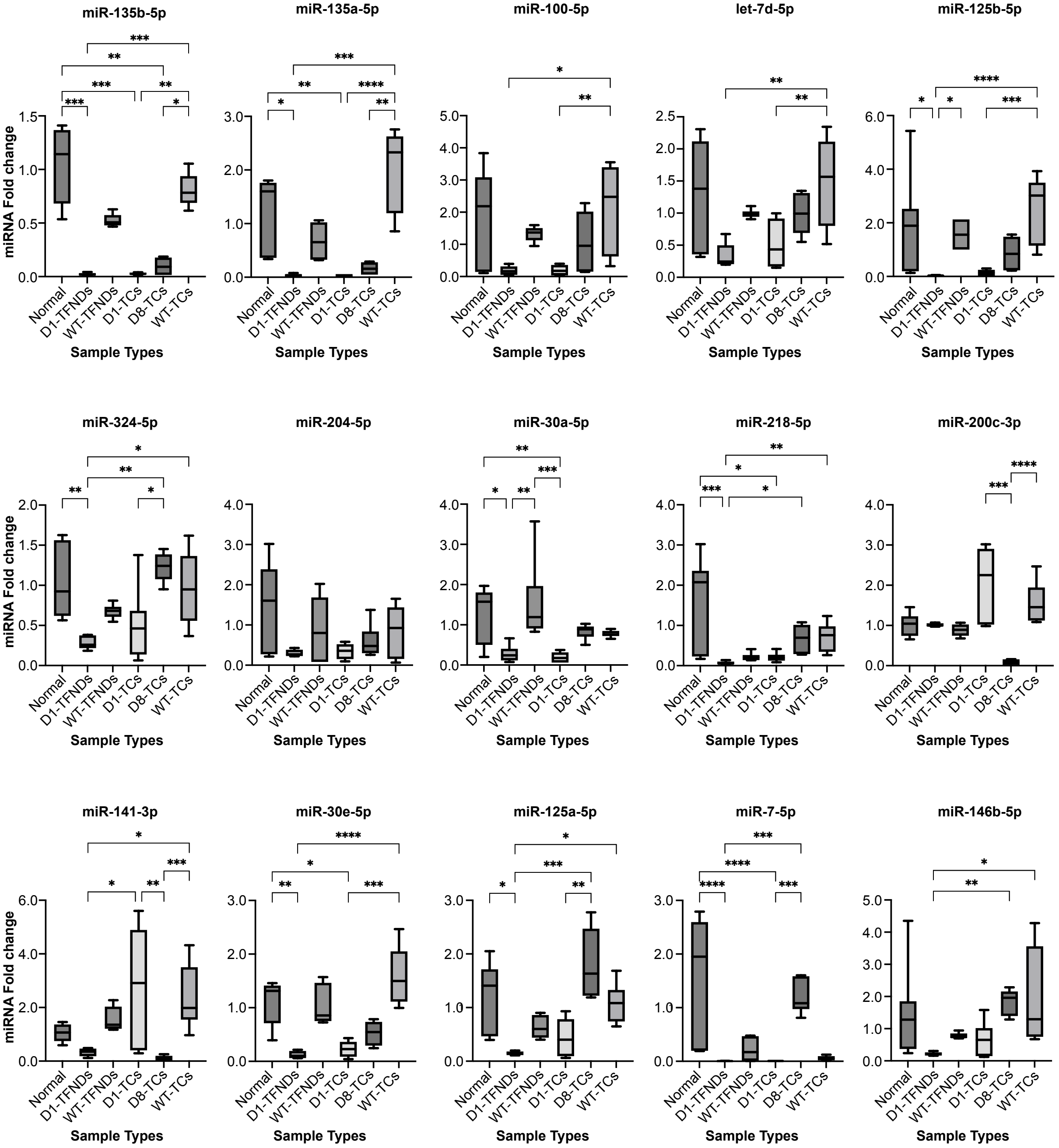

Expression of 15 selected miRNAs in 3 normal thyroid tissues, 2 *DICER1*-mutated thyroid follicular nodular diseases (D1-TFNDs), 3 *DICER1*-mutated thyroid cancers (D1-TCs), 2 DGCR8-mutated TCs (D8-TCs), 2 wildtype (non-*DICER1*/non-*DGCR8*) TFNDs, and 4 wildtype (non-*DICER1*/non-*DGCR8*) TCs for miR-135b-5p, miR-135a-5p, miR-100-5p, let-7d-5p, miR-125b-5p, miR-324-5p, miR-204-5p, miR-30a-5p, miR-218-5p, miR-200c-3p, miR-141-3p, miR-30e-5p, miR-125a-5p, miR-7-5p and miR-146b-5p. Multiple nonparametric pairwise comparisons were performed across the 6 groups. P-values were adjusted using the Dunn’s test, with a significance threshold set at p-value <0.05.

Figure S4: WNT signaling in the PanCK+ component of the *DICER1*-mutated poorly differentiated thyroid carcinoma

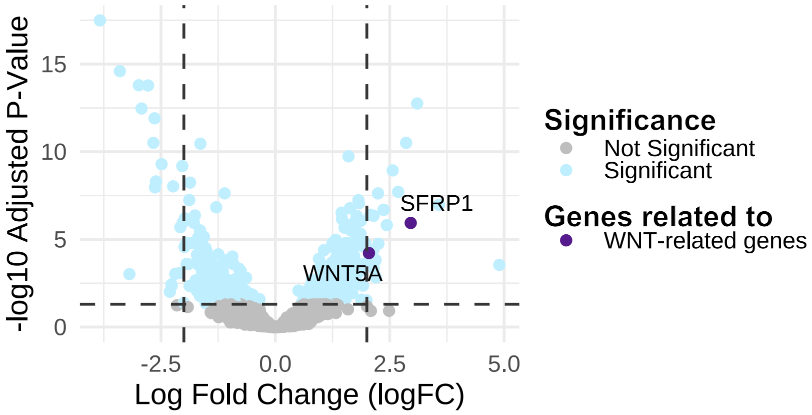

Volcano plot representation of differentially expressed genes observed in the PanCK+ expressing cells of the *DICER1*-mutated poorly differentiated thyroid carcinoma (PDTC) compared to normal tissue. *SFRP1* and *WNT5A* were significantly upregulated (FDR<0.05, logFC>|2|) and associated with WNT signaling (purple dots). Blue dots represent significant differentially expressed genes (FDR<0.05). Grey dots represent gene expression without significant differences.

Figure S5: Expression levels of tumor progression genes in the VIM+ component of the *DICER1*-mutated poorly differentiated thyroid carcinoma

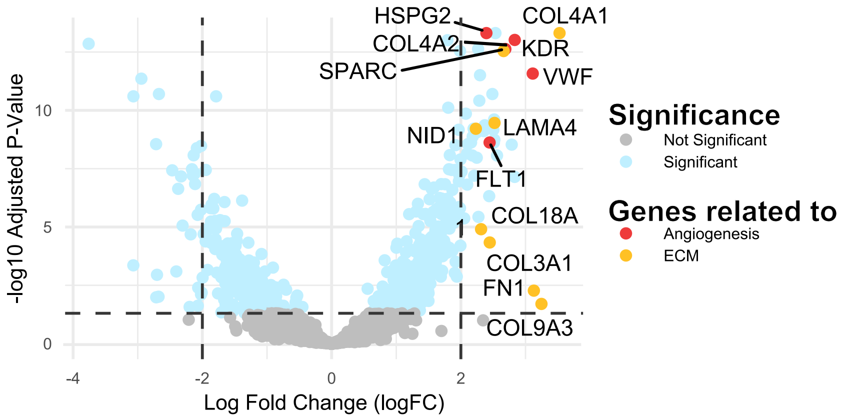

Volcano plot representation of differentially expressed genes, that are targeted by >1 differentially expressed miRNA, in the VIM+ expressing cells of the *DICER1*-mutated poorly differentiated thyroid carcinoma (PDTC) compared to normal tissue. We observed upregulation (logFC>|2|) of genes involved in angiogenesis (seen in red) and extracellular matrix (ECM) (seen in yellow), supporting processes involved in tumor progression. Blue dots represent significant differentially expressed genes (FDR<0.05). Grey dots represent gene expression without significant differences.

Figure S6: Upregulation of immune-related genes in the *DGCR8*-benign and malignant lesions at the region of interest level (PanCK+/VIM+)

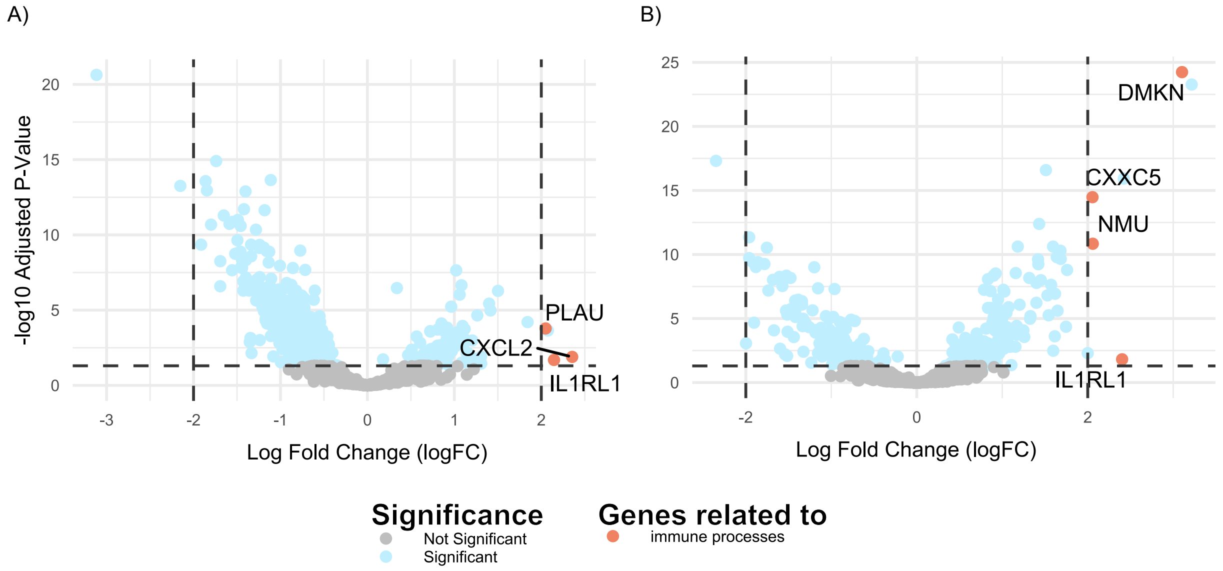

Volcano plot representation of differentially expressed genes at the region of interest level (PanCK+/VIM+) in A) the *DGCR8*-mutated benign lesion (non-neoplastic nodular areas) and B) the *DGCR8*-mutated micro-papillary thyroid carcinoma (microPTC), compared to normal tissue. We observed significant upregulation (FDR<0.05, logFC>|2|) of genes related to immune processes in both (seen in orange). Blue dots represent significant differentially expressed genes (FDR<0.05). Grey dots represent gene expression without significant differences.

Figure S7: Normal thyroid tissue with a *DGCR8* E518K mutation is associated with inflammatory processes **r**

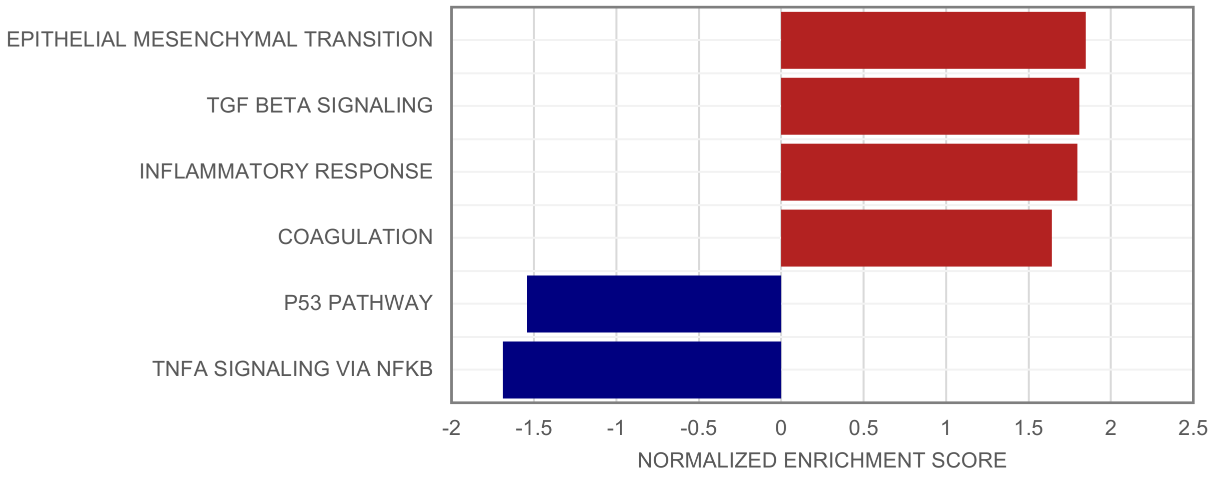

Gene set enrichment analysis (GSEA) comparing normal thyroid tissue with and without a *DGCR8* E518K mutation. Bar charts of GSEA normalized enrichment scores (NES) showing the expression changes between *DGCR8*-normal vs wildtype-normal using the Molecular Signatures Database (MSigDB) Hallmark gene sets. Shown are the NES with a FDR<0.05.

Figure S8: Global mean methylation by CpG location of normal thyroid, non-mutant thyroid cancers and mutant benign and malignant lesions

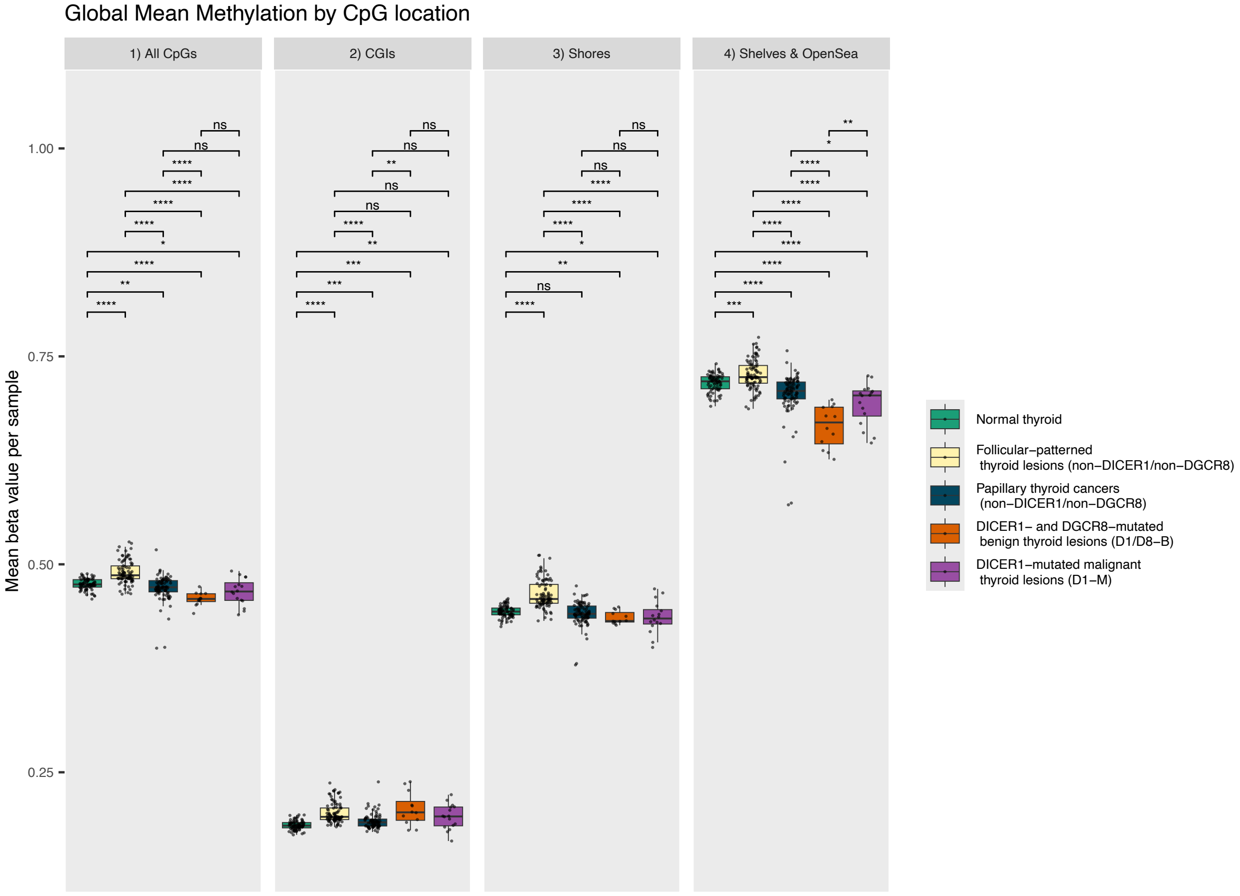

Boxplots representing the global mean methylation levels in normal thyroid, follicular-patterned thyroid lesions (non-*DICER1*/non-*DGCR8*-mutated), papillary thyroid cancers (non-*DICER1*/non-*DGCR8*-mutated), *DICER1*- and *DGCR8*-mutated benign thyroid lesions (D1/D8-B) and *DICER1*-mutated malignant thyroid lesions (D1-M) using 1) all CpGs, 2) CpGs only located in the CpG islands (CGI), 3) CpGs only located in the CpG shores, and 4) CpGs only located in the CpG shelves and the open sea. Statistical significance for differences in global methylation levels between groups was assessed using the Wilcoxon rank-sum test.

Figure S9: Global mean methylation by CpG location of normal thyroid, non-mutant thyroid cancers and mutant benign and malignant lesions

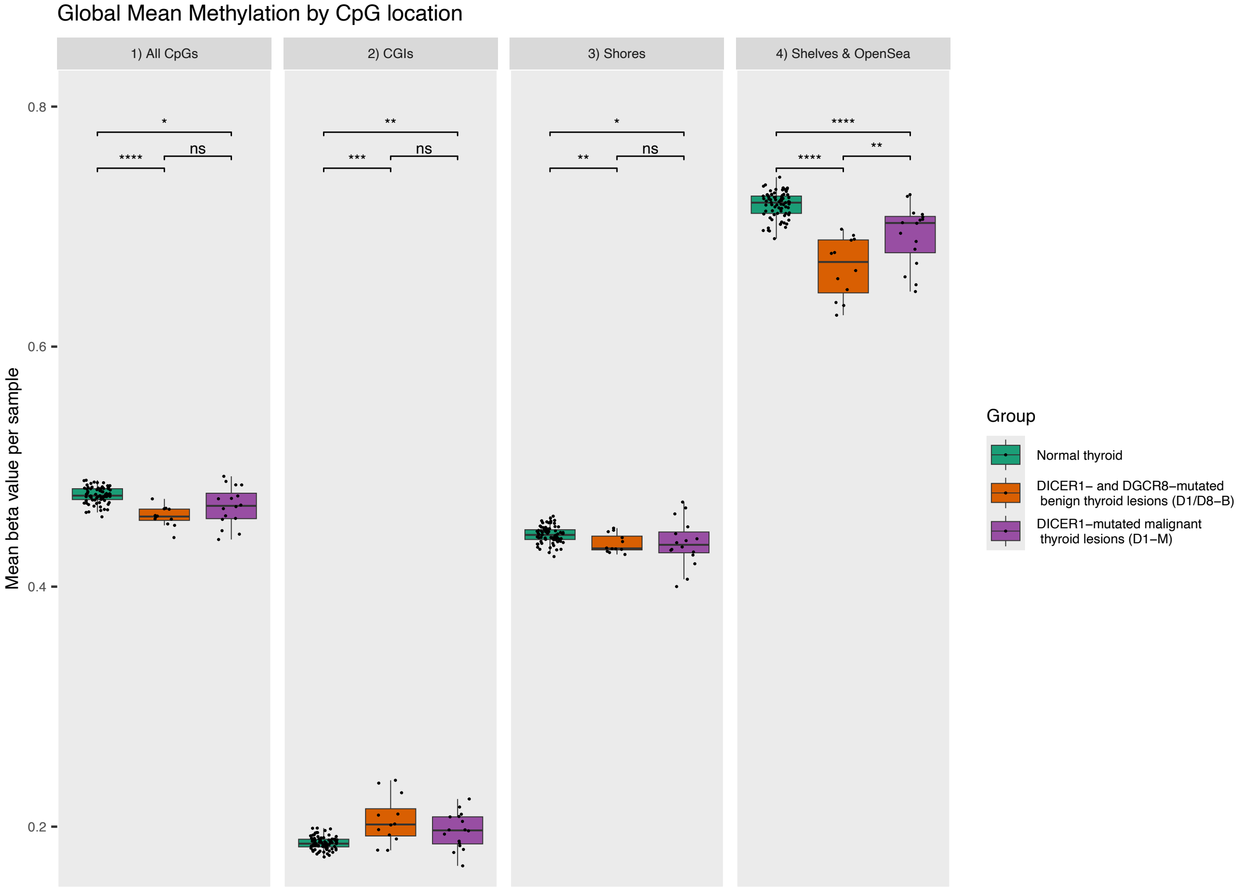

Boxplots representing the global mean methylation levels in normal thyroid, *DICER1*- and *DGCR8*-mutated benign thyroid lesions (D1/D8-B) and *DICER1*-mutated malignant thyroid lesions (D1-M) using 1) all CpGs, 2) CpGs only located in the CpG islands (CGI), 3) CpGs only located in the CpG shores, and 4) CpGs only located in the CpG shelves and the open sea. Statistical significance for differences in global methylation levels between groups was assessed using the Wilcoxon rank-sum test.

SUPPLEMENTAL METHODS

Sample processing

Tumoral DNA from 129 fresh frozen tissue (FFT) samples was isolated with the DNeasy Blood and Tissue Qiagen kit (QIAGEN, Hilden, Germany) and tumoral DNA from 17 archived formalin-fixed, paraffin-embedded (FFPE) material was isolated using the QIAamp DNA FFPE Tissue Kit (QIAGEN, Hilden, Germany). Samples for which tumoral DNA was already extracted and for which only limited DNA was available (N=304), were whole genome amplified using the Genomiphi™ V2 DNA Amplification Kit (Cytiva, Marlborough, MA, USA) and the amplification product was further purified by EDTA/sodium acetate/ethanol precipitation.

Total RNA was extracted from FFT samples using the mirVana™ miRNA Isolation Kit (Thermo Fisher Scientific, Waltham, MA, USA) and total RNA was isolated from FFPE samples using the miRNeasy FFPE kit (Thermo Fisher Scientific, Waltham, MA, USA). Samples were quantified using the Qubit™ RNA High Sensitivity Assay (Thermo Fisher Scientific, Waltham, MA, USA).

Screening of miRNA biogenesis genes

Only hotspot mutations reported in the literature in more than 2 tumors were selected to be screened. four hundred and forty-eight samples were screened for 22 *DICER1*, 1 *DGCR8* and 7 *DROSHA* hotspot mutations and screened for *XPO5* p.R440* using custom Kompetitive Allele Specific PCR (KASP™) genotyping assays (LGC Biosearch Technologies, Hoddesdon, UK). A detailed list of the screened hotspot mutations can be found in Table S18. All identified hotspot mutations in the samples were validated by Sanger sequencing (Table S19).

Another 133 thyroid samples were screened externally by the Hereditary Cancer Group at the Centro Nacional de Investigaciones Oncológicas (CNIO) for *DICER1* mutations using a customized targeted sequencing panel for research of 155 genes with the Roche Kapa HyperPlus technology. This panel includes canonical thyroid cancer drivers and other genes related with thyroid cancer progression. *DGCR8* hotspot mutations were screened by means of PCR amplification and subsequent Sanger sequencing of the amplified amplicon.

Whole exome sequencing

Library preparation

Briefly, the KAPA HyperExome Probes (Roche) were used together with the KAPA HyperCap DS Human mtDNA Design of the KAPA HyperChoice Probes (Roche) to perform whole exome and mitochondrial DNA (mtDNA) enrichment. Short-insert paired-end libraries for bigenomic capture were prepared using the KAPA HyperCap Workflow v3.0 (Roche). The libraries were prepared starting with 100 ng of genomic DNA and using Illumina platform-compatible adapters with unique dual indexes and unique molecular identifiers (Integrated DNA Technologies). The captured libraries were then quality controlled for concentration and size using the Agilent Bioanalyzer DNA 7500 chip. The libraries were sequenced on the NovaSeq 6000 (Illumina) in paired-end mode with a read length of 2x151 bp, following the manufacturer's protocol for dual indexing. Image analysis, base calling, and quality scoring of the run were processed using the manufacturer's software Real Time Analysis (NovaSeq 6000 RTA 3.4.4).

WES alignment and variant calling

After sequencing, reads were mapped to the human genome (hs37d5) using the BWA-mem[1] with default parameters. Alignment files (BAM format) containing only properly paired, uniquely mapping reads were processed using picard tools version 1.110 (http://broadinstitute.github.io/picard/) to add read groups and remove duplicates. The Genome Analysis Tool Kit (GATK)[2] was used for local indel realignment and base recalibration. Processed BAM files were used for the following experiments. For germline variants, HaplotypeCaller (from GATK version 4.1.9.0) with default parameters, was used for single nucleotide variants (SNVs) and small insertion and deletions (INDELS). For somatic SNVs and INDELS, when control samples were available, Strelka (version 2.9.9) and Mutect2 (from GATK version 4.0.8.1) were used. Both programs were used with default parameters. Each program tags high quality variant as "PASS" based on internal quality metrics. The union of the PASS variants from both programs was used for the analysis. When control samples were not available only Mutect2 was run in "tumor-only" mode. To further discriminate

somatic from germline variants two sets were provided to Mutect2: (1) the aggregate variants from a “panel of normals” of 400 individuals, and (2) a set of human variants from gnomAD (https://gnomad.broadinstitute.org) as included in the GATK suit. The “PASS” variants as tag by the Mutect2 suit were used for the analysis. Functional annotations were added to the resulting VCF file using snpEff[3] with the gene annotation obtained from ENSEMBL (http://www.ensembl.org/, version 75).

WES variant filtering and prioritization

To prioritize variants found in the WES, variants seen in a 664 genes list were investigated further. This non-redundant 664 genes list is composed of 152 cancer susceptibility genes (CSGs) from Huang et al.[4], 468 cancer driver genes from Memorial Sloan Kettering Cancer Center (MSKCC)[5], 77 fFND candidate genes from curated literature[6-11] and 112 genes from the Thyroseq v3[12] panel. Those variants which most likely damage the protein (nonsense, canonical splice-site, coding indels and missenses) were considered for further analysis following an *in-house* designed pipeline. To remove common variants and false positive calls, candidate mutations were subjected to several filtering steps and eliminated if they fulfilled any one of the following criteria: (i) genomic position of variant covered by <20 reads, (ii) <5 reads supported by the alternative variant, (iii) variant had allelic ratio <10% for SNVs or <15% for indels, (iv) variant had allele frequency >0.001 in the gnomAD, ExAC, “Grand Opportunity" Exome Sequencing Project (GO-ESP), or The Gene Partnership (TGP) databases. The variants were further filtered following an in-house pipeline using and pathogenicity predictor tools (MetaRNN, REVEL, BayesDel, AlphaMissense, CADD) and the ACMG variant classification. Finally, the Integrative Genomics Viewer was used for the manual examination and visualization of all potential candidate variants.

Copy number variants were predicted using Control-FREEC[13]. A baseline created with the previously mentioned panel of normals was used as a control when a control from the same patient was not available.

***RET* fusion and *TERT* promoter alteration screening**

DICER1- and *DGCR8*-mutated samples for which FFT-derived RNA was available (N=31) were screened for the following 3 RET fusions (*CCDC6::RET, NCOA4::RET, KIF5B::RET*) with an RNA input of approximately 100 ng using the QFusion™ RET Fusion Detection Kit (DiaCarta, Inc, Pleasanton, CA, USA) according to the manufacturer’s protocol. The samples were RT-qPCR amplified on a LightCycler 480 II instrument (Roche Diagnostics, Basel, Switzerland). All *DICER1*- and *DGCR8*-mutated samples were screened for *TERT* promoter alterations by PCR amplification and subsequently Sanger sequenced (Table S19).

miRNA profiling experiment

Briefly, 100 ng of purified total RNA was used for miRNAs sample preparation, which involved miRNAs tagging using an annealing, ligation, and purification protocol. Subsequently, the miRNAs codeset hybridization protocol was employed with denatured samples hybridized with reporter and capture probes at 65 °C for 16 hours. The processed samples underwent purification of the hybridized targets and attachment to the cartridge using the nCounter Preparation Station for imaging via the nCounter Digital Analyzer (CCD camera). Barcodes for each target molecule were counted across a maximum resolution of 555 fields of view (FOV). The code set encompassed 798 mature miRNAs based on miRbase v22.1, along with six positive controls, eight negative controls, six ligation controls, five spike-in controls, and five mRNA housekeeping controls. Initial data quality control and extraction of raw data were carried out using nSolver Analysis Software v4.0 (NanoString Technologies). In-depth quality control and normalization were performed in the RStudio environment. Moreover, as two different versions were used, only probes with the same calibration factor were used for subsequent analysis.

miRNA profiling quality control, sample cleaning and batch correction

Variations in chip versions (v3a and v3b), experiment run dates, and the sample source were recognised as potential confounding factors and were accounted in subsequent bioinformatic analyses. Implementation of technical quality control measures, evaluation of housekeeping genes, and visual inspection of data for identification and removal of poor-quality samples were conducted following the recommendations by Bhattacharya et al.[14]. Quality control indicators for assessing data quality encompassed imaging data, binding density, linearity of positive controls, and limit of detection (LOD). For a more stringent quality control, samples with more than 500 endogenous genes and below 10 raw counts were excluded.

Checking for the removal of known technical factors already identified in the raw expression data and the presence of key biological variation was done using linear mixed model to measure the variation in gene expression associated with individual factors or technical variables. In addition, principal component analysis (PCA) with density plot per component was used to explore the variance structure of all samples before and after Combat-seq[15] batch effect adjustment tool. To avoid potential issues in the differential expression analysis, such as over-adjusted batch effects, loss of statistical power, and inability to detect variables associated with the treatment effect, we chose to fit a linear model using the batches of interest rather than correcting the original data.

RT-PCR and qPCR validations

Sixteen differentially expressed miRNAs from the miRNA profiling experiment were selected to validate by quantitative RT-PCR. cDNA was synthesized from total RNA extracted from/of 41 samples with the Mir-X miRNA First Strand Synthesis kit (Takara Bio, San Jose, CA, USA) according to the manufacturer's instructions. The miRNA expression was measured with the TB Green Advantage qPCR premix (Takara Bio, San Jose, CA, USA), in accordance with the manufacturer's protocol and using custom Forward primers for each miRNA of interest, and a mRQ 3' Primer (a universal reverse primer supplied in the kit). Transcript levels were normalized to U6.

Spatial transcriptomics profiling

GeoMx™ digital spatial profiling was performed as per the supplier's protocol using the human Whole Transcriptome Atlas probe set with read-out by next-generation sequencing (Nanostring Technologies). Briefly, 5 µm FFPE sections of a sporadic DICER1-thyroid case and a germline DGCR8- thyroid case, were processed through deparaffinization, antigen retrieval (Tris-EDTA ph9 for 20 min at 98°C), and Proteinase K digestion (1 ug/mL Proteinase K for 15 min at 37°C) prior to overnight incubation at 37°C with the human Whole Transcriptome Atlas diluted in hybridization Buffer R. The next day, slides were washed twice in 2XSSC/formamide (25 min at 37°C), blocked with Buffer W (30 min at RT) and incubated with an antibody cocktail consisting of PanCK-AF532, CD45-AF594, VIM-AF647, and SYTO13 for 1 hr in a humidity chamber. Slides were washed 3 times in 2XSSC and then scanned on the GeoMx™ DSP instrument (at 20X), after which regions-of-interest (ROIs) in each case were laid down to capture different histological components in triplicates at least. For the DICER1-case, this included a tumour core from a DICER1-mutated PDTC, the tumor's capsule, a non-neoplastic nodular area (DICER1-wildtype benign thyroid lesion) and normal area (DICER1-wildtype). For the DGCR8-case, this included a tumour core from a DGCR8-mutated microPTC, a normal adjacent area, non-neoplastic nodular areas (DGCR8-mutated benign thyroid lesions) and normal area (DGCR8-mutated). A total of 51 ROIs were selected and were segmented further into PanCK+ and/or VIM+ areas, and Whole Transcriptome barcodes were released from these distinct populations by UV light illumination and microfluidic collection. The UV-released barcodes from each of the 84 area-of-illuminations (AOIs) were PCR-amplified to add unique dual-index Illumina i5 and i7 indices and bead-purified twice using AMPure XP beads (Beckman Coutler, A63881). Pooled libraries were sequenced on two 75-cycle High Output flowcells on an Illumina NextSeq sequencer, generating 916M unique raw reads. FASTQ files were converted to DCC files and uploaded onto the GeoMx™ DSP for trimming, stitching, aligning, and deduplication. Targets that were not expressed above the Limit of Quantification in at least 5% of the AOIs were filtered out and data was upper-quartile normalized within each AOI for downstream analysis. In total, 12,236 targets (out of a total of 18,942) were included in the analysis.

Spatial transcriptomics quality control and normalization

Fastq files containing sequencing of UV-cleaved probes were processed into Digital count conversion (DCC) files using the Nanostring GeoMx NGS Pipeline v2.3.3.10. The DCC files were imported into R v4.3.0 for further analysis. Using GeomxTools v3.3.0[16], areas of Illumination (AOI) were assessed for sequencing quality. AOIs retained for analysis if they met the following criteria: Minimum reads per AOI greater than 1000, the percentage of trimmed reads, stitched reads and aligned reads all greater than 80%, the sequencing saturation greater than 50%, the minimum count of negative control probes greater 0.5, No Template Control (NTC) counts greater than 1300, number of nuclei per AOI is greater than 100, the area per AOI is greater than 1000 µm[17], and greater than 5% of genes detected above limit of quantification (LOQ, mean of negative control counts + 2 standard deviation). 6 AOIs were discarded for not meeting the

criteria, for a total of 78 AOIs. Genes were retained in the analysis if detected above LOQ in at least 5% of AOIs for a total of 12236 genes. Counts were normalized using the quartile 3 (Q3) method.

Integration of the miRNA and mRNA data

First, the target gene network (experimentally validated targets only) for the differentially expressed miRNAs was analyzed using miRnet website (https://www.mirnet.ca) through the miRTarBase v9.0 database. Then, we selected those genes that are experimentally validated targets of miRNAs and expressed in our spatial transcriptomic profiling experiment and subsequently analyzed the intersection of these genes with known metabolic pathways to identify relevant biological processes. The intersection represents the number of common genes between our gene lists and each metabolic pathway of the Molecular Signatures Database (MSigDB).

To determine the significance of these intersections, the Fisher's exact test was employed for each statistical test. In that sense, a low p-value indicated that the overlap was not likely due to chance, suggesting a meaningful association between the differentially expressed genes and the cancer hallmark genes in MSigDB.

To mitigate the risk of false positives arising from multiple comparisons, corrections for multiple testing was applied using the false discovery rate approach[127]. This approach allowed us to confidently identify pathways that were significantly enriched in our datasets and could be critical to understanding the underlying biological mechanisms.

DNA Methylation

Thyroid lesion-specific genome-wide DNA methylation profiling was investigated in 3 FFT and 9 FFPE samples (5 TFNDs, 4 FTCs, 1 FVPTC, and 2 PDTCs) using the Illumina HumanMethylation EPIC array at GenomeScan (Leiden, The Netherlands). The data from these samples was combined with data from 5 publicly available datasets from the Gene Expression Omnibus (GEO): GSE121377, GSE197860, GSE97466, and GSE77804 and GSE214568 (thyroid data only); and 1 publicly available dataset from ArrayExpress: E-MTAB-10906. All 6 datasets employed Illumina platforms, with four using the EPIC array and two using the Illumina 450K array. Four datasets were also derived from FFT samples while the other two datasets were derived from FFPE samples. From these 6 studies, we included a total of 277 thyroid samples: 21 FVPTCs; 4 PTC, not otherwise specified (PTC-NOS); 6 ATCs; 6 OTAs; 8 OTCs; 60 CPTCs; 57 normal thyroid tissues; 29 TFNDs; 20 FTAs; 8 tall-cell variant PTC; 26 FTCs; 3 PDTCs; 7 NIFTPs; and 22 nodular hyperplasias. Additionally, we included 2 *DICER1*-mutated FVPTCs and 2 *DGCR8*-mutated FVPTC from the TCGA-THCA project[19]. Of the total 281 samples, 47 samples had available molecular data (*DICER1*, N=26; *DGCR8*, N=8; *BRAF* N=4; *HRAS* N=3; and *NRAS*, N=6).

All methylation analyses were conducted in R (version 4.4.1). Illumina450K, EPICv1 and EPICv2 array data were merged and preprocessed using the minfi package (version 1.52.0). Single-sample Noob normalization was performed. Probes with poor performance were excluded based on detection p-values prior to downstream analyses. Additionally, probes targeting the X and Y chromosomes, those containing single nucleotide polymorphisms (SNPs), and cross-reactive probes were filtered out. The data were further corrected for material type (FFPE/frozen) and array type (450K/EPIC) using the limma package (version 3.62.0). Multidimensional Scaling (MDS) plots were examined to assess potential batch effect.

We calculated mean β-values to explore global methylation levels and investigated cluster-specific and tumor-specific patterns. We focused on CpGs outside of CpG islands (CGI) due to their high density: CpG island shores (0-2kb from CGI), shelves (2-4kb from CGI), and open sea regions (> 4kb from CGI). Group differences were assessed using Wilcoxon rank-sum tests (two-group comparisons) with significance threshold $\alpha=0.05$.

For unsupervised analysis, the UMAP technique (umap package, version 0.20.10.0) was employed for dimensionality reduction. We used 2,279 out of the 2,434 probes (removed low quality probes) described by Marczyk et al. as the features of their classifier algorithm for methylation subtype assignment. Unsupervised hierarchical clustering was performed using the same subset of probes, and the 250 most variably methylated CpGs across the dataset were selected for visualization using the median absolute

deviation. Heatmap visualizations were generated with ComplexHeatmap (version 2.22.0) and dendextend (version 1.19.0) packages. Distance between samples was calculated using Euclidean distances and Ward's linkage method. Subsequent differential methylation analyses were carried on the clusters identified in the heatmap.

Multiple pairwise comparisons were performed across the full set of probes for differential methylation analyses. Differentially methylated positions (DMPs), differentially methylated genes (DMGs) and regions (DMRs) were identified through group-wise comparisons using Champ package (version 2.38.0) and the Probelasso method. Genes were annotated considering probes located in the following regions: 1bp to 1,500bp upstream of the transcription starting site (TSS1500), 5' untranslated region (5'UTR) and the first exon (1stExon). Probes located in 3' untranslated region (3'UTR) and gene bodies were excluded.

P-values were adjusted using the Benjamini–Hochberg method, with a significance threshold set as an adjusted p-value < 0.05 and a beta-value difference > |0.2| for the DMPs and DMGs. DMRs required a minimum of 5 significant probes per lasso (adjusted p-value < 0.05).

Gene set and pathway analyses were conducted using robust rank aggregation applying both overrepresentation (ORA) and gene set enrichment analysis (GSEA) (methylGSA package version 1.24.0 and FGSEA package version 1.32.0) for the GO Biological Processes, KEGG and Hallmarks gene sets. P-values were adjusted using the Benjamini–Hochberg method, with a significance threshold set as an adjusted p-value < 0.05. Genes were annotated using the Bioconductor annotation package IlluminaHumanMethylationEPICanno.ilm10b4.hg19 (version 0.6.0). Probes were selected based on the promoter2 group argument in methylGSA, which includes CpGs located in "TSS1500", "TSS200", "1stExon", or "5'UTR" gene regions.

Supplemental Results

Impact of the miRNA production impairment on the expression landscape of mutant benign and malignant thyroid lesions: In the case of *DICER1*-mutated lesion, given limited statistical power (only 2 *DICER1*-PDTCs in the miRNA analysis) and the absence of a *DICER1*-mutated BTL in the spatial transcriptomic data, we approximated miRNA–mRNA integration. We used the DEGs from the *DICER1*-PDTC (spatial transcriptomic data) compared to normal thyroid tissue and the DEMs (n=39) observed in *DICER1*-thyroid cancers (both differentiated and PDTCs) compared to wildtype thyroid cancers. In this setting, significant associations to pathways related to ECM remodelling (EMT and angiogenesis), proliferation (mitotic spindle and MYC targets 1) and cancer (PI3K/AKT/mTOR signalling, Hedgehog signalling, WNT signalling) were found (Table S13).

SUPPLEMENTAL REFERENCES

1. Li H, Durbin R. Fast and accurate short read alignment with Burrows-Wheeler transform. *Bioinformatics*. Jul 15 2009;25(14):1754-60. doi:10.1093/bioinformatics/btp324
2. McKenna A, Hanna M, Banks E, et al. The Genome Analysis Toolkit: a MapReduce framework for analyzing next-generation DNA sequencing data. *Genome Res*. Sep 2010;20(9):1297-303. doi:10.1101/gr.107524.110
3. Cingolani P, Platts A, Wang le L, et al. A program for annotating and predicting the effects of single nucleotide polymorphisms, SnpEff: SNPs in the genome of Drosophila melanogaster strain w1118; iso-2; iso-3. *Fly (Austin)*. Apr-Jun 2012;6(2):80-92. doi:10.4161/fly.19695
4. Huang KL, Mashl RJ, Wu Y, et al. Pathogenic Germline Variants in 10,389 Adult Cancers. *Cell*. Apr 5 2018;173(2):355-370.e14. doi:10.1016/j.cell.2018.03.039
5. Zehir A, Benayed R, Shah RH, et al. Mutational landscape of metastatic cancer revealed from prospective clinical sequencing of 10,000 patients. *Nat Med*. Jun 2017;23(6):703-713. doi:10.1038/nm.4333
6. Bakhsh AD, Ladas I, Hamshere ML, et al. An InDel in Phospholipase-C-B-1 Is Linked with Euthyroid Multinodular Goiter. *Thyroid*. Jul 2018;28(7):891-901. doi:10.1089/thy.2017.0312
7. Vantyghem MC, Faivre-Defrance F, Marcelli-Tourvieille S, et al. Familial partial lipodystrophy due to the LMNA R482W mutation with multinodular goitre, extrapyramidal syndrome and primary hyperaldosteronism. *Clin Endocrinol (Oxf)*. Aug 2007;67(2):247-9. doi:10.1111/j.1365-2265.2007.02870.x
8. Nishihara E, Hishinuma A, Kogai T, et al. A Novel Germline Mutation of KEAP1 (R483H) Associated with a Non-Toxic Multinodular Goiter. *Front Endocrinol (Lausanne)*. 2016;7:131. doi:10.3389/fendo.2016.00131
9. Rivera B, Nadaf J, Fahiminiya S, et al. DGCR8 microprocessor defect characterizes familial multinodular goiter with schwannomatosis. *J Clin Invest*. Mar 2 2020;130(3):1479-1490. doi:10.1172/jci130206
10. Teshiba R, Tajiri T, Sumitomo K, Masumoto K, Taguchi T, Yamamoto K. Identification of a KEAP1 germline mutation in a family with multinodular goitre. *PLoS One*. 2013;8(5):e65141. doi:10.1371/journal.pone.0065141
11. Yan J, Takahashi T, Ohura T, et al. Combined linkage analysis and exome sequencing identifies novel genes for familial goiter. *J Hum Genet*. Jun 2013;58(6):366-77. doi:10.1038/jhg.2013.20
12. Nikiforova MN, Mercurio S, Wald AI, et al. Analytical performance of the ThyroSeq v3 genomic classifier for cancer diagnosis in thyroid nodules. *Cancer*. Apr 15 2018;124(8):1682-1690. doi:10.1002/cncr.31245
13. Boeva V, Popova T, Bleakley K, et al. Control-FREEC: a tool for assessing copy number and allelic content using next-generation sequencing data. *Bioinformatics*. Feb 1 2012;28(3):423-5. doi:10.1093/bioinformatics/btr670
14. Bhattacharya A, Hamilton AM, Furberg H, et al. An approach for normalization and quality control for NanoString RNA expression data. *Brief Bioinform*. May 20 2021;22(3)doi:10.1093/bib/bbaa163
15. Zhang Y, Parmigiani G, Johnson WE. ComBat-seq: batch effect adjustment for RNA-seq count data. *NAR Genom Bioinform*. Sep 2020;2(3):lqaa078. doi:10.1093/nargab/lqaa078
16. Griswold M, Ortogero N, Yang Z, Vitancol R, Henderson D. GeomxTools: NanoString GeoMx Tools. 2024;doi:doi:10.18129/B9.bioc.GeomxTools

17. Ritchie ME, Phipson B, Wu D, et al. limma powers differential expression analyses for RNA-sequencing and microarray studies. *Nucleic Acids Res*. Apr 20 2015;43(7):e47. doi:10.1093/nar/gkv007
